# Matrix-Associated Gene Expression and Annotated Tumor–Stroma Composition of Transcriptomically Defined Cold Regions in Triple-Negative Breast Cancer

**DOI:** 10.3390/cancers18213476

**Published:** 2026-10-28

**Authors:** William D. Lituma-Gonzalez, Amogh V. Arunprasad, Hugo Chancay, Varshithaa Jayavelan, Meenakshi Chakravadhanula, Santiago Ballaz, Shanmugamurthy Lakshmanan

**Affiliations:** 1 Department of Transdisciplinary Science & Technology, Siddha Vetha Multiversity, Newark, NJ 07103, USA; william.lituma@yachaytech.edu.ec (W.D.L.-G.); amogh.v.arunprasad@siddhavethamultiversity.org (A.V.A.);; 2 NFInnovations, Renton, WA 170129, USA; hugo.chancay@nfinnovations.com; 3 Centro de Investigación Biomédica (CENBIO), Facultad de Ciencias de la Salud Eugenio Espejo, Universidad UTE, Av. Mariscal Sucre s/n y Mariana de Jesús, Quito 170129, Ecuador

**Keywords:** triple-negative breast cancer, spatial transcriptomics, extracellular matrix, cancer-associated fibroblasts, tumor microenvironment, spatial heterogeneity, cell-type deconvolution

## Abstract

Background/Objectives: Regional transcriptomic labels can reflect cell mixtures and expression states. We examined how transcriptomically defined cold regions in triple-negative breast cancer relate to matrix-associated expression, annotated composition, spatial neighborhoods, and cell-type attribution. Methods: We reanalyzed public data from 43 discovery sections (22 patients) and 11 cancer captures of a secondary dataset with discovery-derived labels. Computational spot labels included desert (D), excluded (E), inflamed (I), and ambiguous-cold. Hierarchical models estimated E−D contrasts and E−I gene contrasts with and without sequencing-depth and estimated tumor-abundance adjustment; tests underwent Holm correction. Further analyses addressed annotated composition, neighborhoods, and pooled exploratory cell-type attribution. Results: Estimated cancer-associated fibroblast (CAF) abundance was higher in E- than D-labeled regions (standardized within-section effect 0.78; 95% confidence interval 0.55–1.00), as was expression of PDGFRB, COL3A1, COL5A1, and COL6A1; the three collagen genes remained higher than in D- and I-labeled regions after adjustment. Across 11 captures, the median within-capture difference in tumor-annotated spot fraction (E minus D) was −14.1 percentage points (95% bootstrap interval −33.5 to −10.7; nine negative, two ties; interval reached zero without in situ annotations). In discovery, 330 of 822 ambiguous cold spots neighbored both D- and E-labeled spots, exceeding two null models. Pooled models estimated positive COL3A1 and COL5A1 differences within CAFs; immune readouts depended on scoring and adjustment. Conclusions: We characterized a stromal–transcriptomic contrast: E-labeled regions showed higher matrix-associated expression and estimated CAF abundance than D-labeled regions and, in the secondary dataset, lower annotated tumor fraction. These measurements refine interpretation without implying functional immune exclusion.

## Simple Summary

Maps of gene activity show where signals occur in a tumor, but each measured spot can contain several cell types, so signals may reflect cell mixture as well as gene activity. We reanalyzed public maps from triple-negative breast cancer, a subtype of breast cancer, comparing two types of regions that gene activity marked as immune-poor. One type showed more estimated fibroblasts associated with the tumor and stronger activity of genes linked to the surrounding matrix than the other. Spots with uncertain labels lay near both region types more often than chance predicts. In a second dataset, pathology annotations showed that this same type had a smaller proportion of tumor-annotated spots among those annotated as tumor or supporting tissue. Each measurement captures a different property, so they are best read together. These findings can help interpret tumor maps and guide future tissue and single-cell studies.

## 1. Introduction

In triple-negative breast cancer (TNBC), adding anti-PD-1 therapy to chemotherapy increased the pathological complete response rate in early disease [1] and overall survival in PD-L1-positive advanced disease [2]. Lymphocyte presence is only part of the immunologic picture; position relative to tumor cells and stroma also matters. Multiplexed ion beam imaging of 41 TNBCs showed that tumors with similarly sized immune infiltrates differed in whether immune and tumor cells were mixed or compartmentalized, and compartmentalization was associated with longer survival [3]. Across complementary TNBC cohorts, Hammerl et al. defined an excluded phenotype by predominant CD8+ T-cell localization at the tumor border, identified stromal collagen-10 deposition in that phenotype, and linked an excluded gene-expression signature to nonresponse to anti-PD-1 therapy in metastatic disease [4]. Laser-capture expression profiling combined with CD8+ T-cell localization distinguished tumors with tumoral CD8+ infiltration from tumors lacking tumoral CD8+ T cells (fibrotic-stroma signatures) and from tumors with stromal restriction of CD8+ T cells, the latter two with poor outcomes [5]. Multicellular spatial features have been associated with checkpoint-blockade response in a randomized neoadjuvant TNBC trial [6].

Several of these categories are linked to stromal features, yet “stromal” can refer to different properties. Cancer-associated fibroblasts (CAFs) are heterogeneous in breast cancer: the myofibroblastic subsets CAF-S1 and CAF-S4 accumulate differentially in TNBC, and CAF-S1 promotes regulatory T-cell-mediated immunosuppression [7]; CAF-S1 comprises eight clusters, including matrix- and TGFβ-associated programs whose signatures were linked to primary immunotherapy resistance in melanoma and non–small cell lung cancer cohorts [8]; and single-cell data from TNBC resolve a myofibroblast-like and an immunomodulatory CAF state [9]. Because CAF functions include matrix deposition and remodeling and crosstalk with leukocytes [10], four properties should be kept apart: which CAF population or state is present, whether it expresses matrix genes, whether matrix is deposited and organized, and whether it modulates immune cells. In breast cancer, single-cell and spatial transcriptomic data indicated that attributing TGFβ signaling to one CAF population is not enough, because EMILIN1, a TGFβ inhibitor expressed by several CAF populations, was associated with CD8+ T-cell infiltration within TGFβ-rich areas [11]. Matrix-associated RNA programs and histologic immune exclusion are therefore related but distinct measurements.

Spatial transcriptomics retains tissue coordinates while profiling expression [12], so local differences in matrix- and immune-related expression can be examined within a section. In spot-based platforms, however, one measurement can contain RNA from several cells [13]. A regional difference can reflect different numbers of cells of a given type, different expression within the same cell type, or both. Reference-based methods estimate the cell-type composition of each spot [13,14,15], and C-SIDE models cell-type-specific differential expression across tissue contexts [16]. Pathology annotation adds tumor–stroma composition, and the arrangement of labels on the spot graph adds neighborhood organization; each informs a different property, and none is interchangeable with a histologic immunotype.

A key interpretive question is what a transcriptomically defined cold region represents: matrix-associated expression, a difference in tissue composition, a cell-type-conditioned program, or a particular local arrangement. Visium analyses of TNBC have described clusters, including a stromal–immune cluster dispersed from tumor clusters, and interpreted immune content as largely sequestered in the fibrotic compartment [17], and spatial transcriptomics has also described substantial heterogeneity in TNBC with potential clinical implications [18]; such cluster-level descriptions do not show how labels derived from RNA scores relate to matrix-associated expression, annotated composition, and neighborhood organization after accounting for cell mixtures. We examined the regional expression, compositional, and spatial correlates of transcriptomically defined cold-region labels in TNBC, and assessed how their interpretation changed across scoring, composition-adjustment, and cell-type-attribution approaches. Transcriptomic scores assigned operational region labels in the discovery Visium dataset, and hierarchical models compared desert-labeled (D) and excluded-labeled (E) spots within sections; a second public dataset supplied pathology annotations. These labels borrow the vocabulary of image-defined immunotypes [19,20] but denote local transcriptomic states of multicellular spots, not histologic compartments. Two secondary questions followed: whether E-labeled regions differed from D-labeled regions in annotated tumor–stroma composition, and whether regional patterns persisted or changed under cell-type attribution and analytical sensitivities. MYC-associated measurements and gene–drug annotations are reported as exploratory context.

Together, these analyses characterize what we term a stromal–transcriptomic contrast between E- and D-labeled regions in matrix-associated expression and, where annotations exist, annotated tumor–stroma composition. They also examine how this contrast relates to neighborhood organization and model-conditioned cell-type attribution, without equating these measurements with one another or with functional immune exclusion.

## 2. Materials and Methods

### 2.1. Study Design and Data Sources

This observational reanalysis used public datasets; no samples were collected. Discovery data (GSE210616 [17]; 10× Genomics Visium, Pleasanton, CA, USA) comprised 43 sections from 22 patients; patient assignments reconstructed from barcode overlap were reconciled with GEO SOFT metadata. The secondary dataset (GSE213688 [21]) comprised 15 captures and 12 donor labels derived from GEO titles; 11 cancer captures formed the primary population and all 15 a sensitivity population. Existing labels were retained without recalculating thresholds after cancer restriction, providing directional corroboration, not validation of the framework. GSE176078 [22] provided the single-cell reference (15 cell types or states). Populations and units of analysis are summarized in Table 1.

### 2.2. Expression Processing, Operational Labels, and Readouts

Discovery scores and labels were calculated from discovery spots only. Expression readouts used log-normalized raw expression, log [1 + 10^4^ × counts/spot total]; secondary analyses used the log-normalized preprocessed matrix and stored labels.

Classification used within-sample z-standardized Tumor (five genes), CD8 (seven), Silencing (seven) and Barrier (nine) scores with global percentile gates. Tumor below the 60th percentile defined Normal_Stroma; among remaining spots, CD8 above the 75th percentile defined Inflamed (I). Remaining spots were Desert (D) if normalized Silencing − Barrier exceeded +0.10, Excluded (E) if below −0.10, and Ambiguous_Cold otherwise, including both boundaries. Percentiles were used for all spots in the object. Signatures, decision rules and readout definitions are in Supplementary Table S1.

Readouts comprised eight immune panels, estimated CAF abundance, four stromal genes, and MYC mRNA, Hallmark36 and MYC_TF_clean. Discovery immune-panel scores used score_genes in Scanpy [23] (seed 0) alongside mean log-expression, with 20-seed sensitivity analyses; secondary immune readouts used effective-list means. Classifier overlap and an alternative ISG panel were evaluated separately. Labels represent operational transcriptomic states of multicellular spots, not histological immunotypes; MYC scores are expression-derived summaries, not direct measures of transcription-factor activity.

### 2.3. Cell-Type Abundance Estimation and Regional Models

Cell-type abundances were estimated with Cell2location [15]; primary specification A0 used posterior means. Sensitivities were Cell2location A1 (discovery spots only; detection_alpha = 200) and A2 (broader preprocessed population; detection_alpha = 20), both with N_cells_per_location = 30, 30,000 full-batch epochs, learning rate 0.002 and seed 42, and Stereoscope A3 [14], run after filtering spots (≥500 counts, ≥200 genes, <20% mitochondrial) and genes (≥10 spots). A3 proportions were multiplied by 30, omitting Tumor from the full-composition model because of constant-sum dependence. A0 and A2 were fitted jointly on discovery and secondary spots, and A1 on discovery spots only; the E−D CAF-abundance contrast was d_intra 0.777 under A0 and 0.826 under A1 (Supplementary Table S4).

Regional models used REML in statsmodels 0.14.5 [24]. Discovery specification B included group, its section mean, covariates and their section means (Mundlak-type terms [25]), a patient intercept, and independent section-intercept and group-slope variance components (covariance not estimated). Specification A, primary in the secondary cohort and a discovery sensitivity, used correlated capture/section intercepts and slopes without a patient level. Models included no covariates (M0), Tumor (M1), Tumor plus CAF (M2), 15 abundances (M3), principal components (M3-PCA), reduced groupings (M3-RED), and depth-adjusted variants, including M0adj (log(UMI) plus Tumor). Fits required convergence, a finite coefficient and SE, a retained random slope, and positive residual and slope variances after sequential optimization. The standardized effect was d_intra = β/SD_residual from the same fit. Adjustment was statistical conditioning on estimated composition, not causal mediation; spatial covariance was not explicitly modeled. Specifications are in Supplementary Table S2 (Block A; notes a, i).

### 2.4. Statistical Inference and Sensitivity Analyses

Regional coefficients used two-sided Wald tests with Holm correction by family, model, contrast and constructor: eight immune readouts, five E−D readouts, four-gene stromal contrasts, three MYC readouts and four panel comparisons. Non-estimable entries retained their family position through *p* = 1 as a bookkeeping placeholder, not a test result. No prospective sample-size calculation was performed.

The paired spatial bootstrap used 500 draws, resampling patients, sections within patients and contiguous within-section blocks with replacement. Models were refitted with labels, scores, abundances and PCA transformations fixed. Intervals were coefficient percentiles; tail summaries referenced zero without imposing a null distribution. Readout–model combinations with >30% failed draws were non-interpretable.

Sensitivities included leave-one-patient/section-out exclusions, a stromal section bootstrap (sections, not patients), and section-level random-effects (REML) meta-analysis with spatial-block bootstrap standard errors and Hartung–Knapp adjustment [26], treating sections as independent. Label analyses used threshold sweeps, gene dropout, permutations and alternative configurations; Leiden [27] concordance across preprocessing variants used within-section permutation nulls. Parameters, units and rules are in Supplementary Table S2 (Block B; notes b, c).

### 2.5. Pooled Cell-Type-Specific Expression

C-SIDE [16] modeled pooled E/D spots (E = 1, D = 0) in four related arms using RCTD [13] or A0 weights, each with and without Section 38 [13], without patient or section terms. Counts for 36 exported genes were reconstructed from log-normalized raw using estimated spot totals. Estimable pairs required finite σ (natural-log E/D fold change), BH-adjusted q and SE < 10; Holm used original *p*-values within gene family, arm and primary/secondary cell-type set. These analyses provided model-conditioned attribution, not patient-level replication or proof of intrinsic expression. Settings and spot counts are in Supplementary Table S2 (note e).

### 2.6. Annotated Composition and Spatial Neighborhoods

Discovery neighborhoods used six nearest Euclidean neighbors per section. The global statistic was the proportion of Ambiguous_Cold spots, pooled across sections, with at least one D and one E neighbor, compared against two nulls (within-section label permutation; rigid relocation of contiguous ambiguous components onto existing sites; 999 replicates each). Intermediacy compared ambiguous-spot means with D- and E-interior means across six measurements using strict inequalities (criteria in Supplementary Table S2).

In cancer captures, P9 calculated the tumor/(tumor + stroma) spot fraction f per label (≥10 such spots). The capture-level contrast δ = f(E) − f(D) was summarized by its median, a 10,000-draw capture bootstrap and an exact two-sided sign test excluding ties; saturated captures were retained. A separate donor-level analysis (one capture per evaluable donor; interface dictionary distinct from P9) compared hexagonal-grid distances to the annotated tumor–stroma interface for Ambiguous_Cold versus pooled D+E spots: AUC counted ties as 0.5, with a median-bootstrap interval and exact sign test against 0.5. Spot fractions were not interpreted as cell fractions or tissue areas. Dictionaries, sensitivities and eligibility rules are in Supplementary Table S2 (Block B; note f).

### 2.7. Pseudo-Spot Stress Test and Drug–Gene Annotation

TNBC reference cells were split by patient into five Ref and five Eval patients. Ref profiles served for verification; pseudo-spots sampled Eval cells with replacement using A0-derived compositions. Type counts were rounded, zero-truncated values of 30 × proportion plus Gaussian noise (SD 0.5), without fixing the total at 30 or thinning to library depth. Scenarios S1–S3 varied composition, source-cell-type expression, or both. M0 and M3 were fitted on raw and normalized scales; simulated M3 used the true realized composition, real-data models the estimated one. Metrics were intervals containing zero (S1), coverage of the average effect on E-like pseudo-spots (ATT; S2), and descriptive comparisons (S3), whose estimands could differ. Design details are in Supplementary Table S2 (Block B; note g).

Drug–gene annotation used existing DGIdb [28] records, queried on 20 August 2026 and audited without new queries. Recorded interaction type preceded gene–drug deduplication; prioritization also used recorded approval, score, and an eight-gene whitelist. Compound-name consolidation was mechanical, and the release version was not recorded. Annotations describe database records, not TNBC approval, therapeutic efficacy or region-specific effects (Supplementary Table S2, note h).

### 2.8. Computational Environments

Analyses used three high-performance computing environments: tnbc_spatial (Python 3.10.18; scvi-tools 1.1.2, cell2location 0.1.5), spatial_tnbc_a (Python 3.10.18), and r_spacexr (R 4.3.3; spacexr 2.2.1). Annotated-composition and drug–gene analyses were run locally. Package versions are in Supplementary Table S2 (note j).

## 3. Results

### 3.1. Study Populations and Label Stability

The discovery cohort comprised 43 sections from 22 patients (49,464 spots); 14,884 entered the primary E−D comparison (5582 E-labeled and 9302 D-labeled; Table 1; Supplementary Figure S1). Secondary analyses used the 11 cancer captures, with labels retained without recalculating thresholds after cancer restriction; all 15 captures formed a sensitivity population. Definitions and models are in Supplementary Tables S1 and S2; intervals are 95% confidence intervals unless stated otherwise.

The reference E−D contrast of the threshold sweep (spot-level d = 0.487; section-paired d_z = 1.207) retained its direction across all 770 re-labeled classifier-threshold configurations, and at least 80% of its baseline magnitude in 99.1% (d) and 79.1% (d_z) of them (Supplementary Figure S2; Supplementary Table S3). Within-section label permutation gave a null d of 0.011 ± 0.013 (SD; *p* = 2.0 × 10^−4^, the minimum attainable with 5000 permutations). Removing 50% of classifier genes lowered exact label agreement to 65.2%, whereas the spot-level effect retained 0.84 of its baseline value. Leiden clustering of 15,706 cold spots agreed little with the D/E labels on globally scaled data (best adjusted Rand index [ARI] over eight resolutions, 0.002) and largely followed patients (ARI 0.972); it met the prespecified signal criterion only after within-section scaling (0.142) or spatial smoothing with Harmony (0.107), each above the 95th percentile of its permutation null (≤0.008; Supplementary Figure S3; Supplementary Table S3).

### 3.2. Regional Stromal and Matrix-Associated Contrasts

Estimated CAF abundance was higher in E- than in D-labeled spots in 42 of 43 sections (paired d_z = 1.207; section-bootstrap interval 0.980–1.627) and 22 of 22 patients (d_z = 1.312; patient-bootstrap interval 1.036–1.895); hierarchical d_intra was 0.777 [0.552, 1.002] (Figure 1A; Supplementary Table S4). Four stromal genes outside the classifier signatures were also higher under the unadjusted model (M0; d_intra 0.450–1.034 across genes); all five readouts passed Holm correction. Adjustment for log UMI and estimated tumor abundance (M0adj) moderated the gene estimates (0.411–0.908), all Holm-significant. Relative to I-labeled spots, COL3A1 (0.298 [0.222, 0.374]), COL5A1 (0.217 [0.144, 0.289]), and COL6A1 (0.168 [0.119, 0.218]) remained higher under M0adj and passed Holm correction, whereas adjusted PDGFRB was small and uncertain (0.121 [−0.107, 0.349]; Holm *p* = 0.299; section bootstrap not interpretable; Figure 1B; Supplementary Table S4). Leaving out each patient changed the CAF d_z by ≤10.7% without sign changes, and M0 estimates for CAF abundance and PDGFRB kept their sign across 12 threshold configurations. In the 15-capture sensitivity set, CAF abundance was higher in E-labeled spots in 14 of 15 captures (d_z = 1.508; bootstrap interval 1.036–2.626).

### 3.3. Model-Conditioned Cell-Type Attribution

C-SIDE yielded pooled, exploratory, cell-type-conditioned estimates from four related arms (RCTD or A0 weights, each with and without Section 38 [13]) that modeled different cell-type sets. Within CAFs, COL3A1 and COL5A1 were higher in E-labeled spots in all arms (σ = 0.208–0.276 and 0.204–0.292, natural-log fold change E versus D; Holm *p* ≤ 1.3 × 10^−22^ and ≤9.2 × 10^−13^; Figure 1C; Supplementary Table S5). PDGFRB coefficients were positive in all arms, with Holm evidence under A0 weights (σ = 0.331 and 0.332; Holm *p* ≤ 3.4 × 10^−5^) but not under RCTD weights (σ = 0.123 and 0.060; Holm *p* = 0.099 and 0.269). COL6A1 coefficients were negative in all arms (σ = −0.111 to −0.056) and passed Holm correction only under A0 weights (adjusted *p* = 0.049); the positive regional COL6A1 contrast was not accompanied by a positive CAF-conditioned estimate.

### 3.4. Annotated Tumor–Stroma Composition

In secondary cancer captures, tumor-annotated spots formed a smaller fraction of tumor- plus stroma-annotated spots in E- than in D-labeled regions (Figure 2A,B; Table 2). Across 11 captures, the median within-capture E−D difference was −0.141 (−14.1 percentage points; 95% capture-bootstrap interval −0.335 to −0.107): negative in nine, positive in none, and zero in two (M13 and M15, without stroma-annotated spots in either label). The two-sided sign test excluding ties gave *p* = 0.0039, the minimum attainable with nine informative captures. Removing in situ annotations gave −0.138 (−0.248 to 0.000; seven negative, four ties), because M8 and M11 then lacked tumor-annotated spots; restricting stroma to tumor-associated stroma and fibrosis gave −0.141 (−0.302 to −0.107; Supplementary Table S6). These variants are alternative definitions of one contrast, not replications, and quantify annotated spots rather than cells or area. With the regional readouts, they describe an operational stromal–transcriptomic contrast between E- and D-labeled regions.

### 3.5. Mixed-Label Neighborhoods and Annotated-Interface Proximity

In discovery, 330 of 822 Ambiguous_Cold spots had at least one D- and one E-labeled spot among their six nearest Euclidean neighbors (ambiguous-spot-weighted fraction 0.401), exceeding the reported null intervals for within-section label permutation (0.302–0.369) and rigid relocation of contiguous ambiguous components (0.226–0.286; *p* = 0.0010 for each, the minimum attainable with 999 replicates; Figure 2C; Supplementary Table S7). In 36 of 43 sections, ambiguous-spot means lay between D- and E-interior means for at least four of six measurements. In the secondary cohort, per-donor AUCs for hexagonal-grid proximity of Ambiguous_Cold versus pooled D/E spots to the annotated tumor–stroma interface centered near 0.5 (median 0.486; bootstrap interval 0.411–0.554; 7 of 11 cancer captures evaluable, one per donor; 221 Ambiguous_Cold spots; sign test *p* = 1.0; Figure 2D). The analyses measured mixed-label neighborhoods and proximity to an annotation-defined interface, respectively.

### 3.6. Regional Immune Readouts

Immune readouts were higher in E- than in D-labeled spots, attenuated after composition adjustment, and depended on panel and constructor. With score_genes and no covariates (M0), seven of eight prespecified readouts passed Holm correction, the largest estimate being HLA-A+B2M (0.438 [0.332, 0.544]; Figure 3A; Supplementary Table S8); STING did not (d_intra = 0.116 [−0.023, 0.255]; Holm *p* = 0.102). After adjustment for 15 estimated cell-type abundances (M3), all estimates decreased, and four remained Holm-significant, led by HLA-A+B2M (0.255 [0.161, 0.348]) and the six-gene composite (0.210 [0.114, 0.307]); STING and ISG10 had nominal intervals excluding zero without passing Holm correction. Alternative adjustments retained positive directions among estimable readouts, but Holm evidence varied (three readouts with a reduced covariate set, none with principal components). Across four deconvolution specifications, the M3-to-M0 ratio of standardized effects for HLA-A+B2M ranged from 0.216 to 0.598, with concordant attenuation for seven of eight readouts (ISG10 differed under Stereoscope; Figure 3B).

Paired panel comparisons were Holm-significant in all four prespecified comparisons under M0 and in two under M3; for example, the seven-gene MHC-I contrast exceeded that of the five-gene panel lacking HLA-A and B2M under M3 (Δ = 0.0281 [0.0142, 0.0421] z-score units; Holm *p* = 3.09 × 10^−4^). With mean log-normalized expression, three readouts passed Holm correction at M0 and two at M3. Control genes sampled by score_genes had lower mean expression in E-labeled spots (d_intra −0.269 to −0.428) and tracked sequencing depth within sections (median Spearman ρ 0.523–0.654); adjustment for log UMI and tumor abundance reduced the E−D difference between constructors by 81–92%, leaving small residuals for the two MHC-I panels. Across 20 control-gene seeds, magnitudes varied without sign reversals among estimable fits (HLA-A+B2M M0, 0.340–0.494). C-SIDE coefficients for HLA-A and B2M were negative in CD8 T cells (A0 weights) and macrophages (RCTD weights; Supplementary Table S5), differing in direction from the positive regional MHC-I contrast.

Section-level meta-analysis (REML, Hartung–Knapp) gave positive mean E−D differences for all eight score_genes readouts (I^2^ 30.2% for STING to 87.7% for HLA-A+B2M; 95% prediction intervals including zero for all; positive differences in 30–39 of 43 sections; Figure 3C). In the 11 secondary cancer captures (capture as unit), all eight readouts had the discovery sign, with similar point estimates for HLA-A+B2M (0.395 [0.108, 0.682] versus 0.371 [0.255, 0.486] in discovery), but none passed Holm correction (HLA-A+B2M Holm *p* = 0.056; Figure 3D). In the 15-capture sensitivity set, HLA-A+B2M passed Holm correction (0.319 [0.113, 0.524]; Holm *p* = 0.019), and only STING had a discordant sign.

### 3.7. Supporting Analyses

MYC-associated measurements were higher in D- than in I-labeled regions under M0 and passed Holm correction: MYC mRNA (d_intra = 0.330 [0.265, 0.395]), Hallmark36 (0.417 [0.303, 0.531]), and the expression-derived MYC_TF_clean score (0.486 [0.331, 0.640]; Supplementary Figure S4; Supplementary Table S9). Hallmark36 and MYC_TF_clean correlated with estimated tumor abundance, and adjustment for it (M1) reduced them to 0.155 [0.058, 0.252] and 0.179 [0.021, 0.338], whereas MYC mRNA changed less (0.285 [0.220, 0.350]); log-UMI adjustment showed the same pattern (Supplementary Table S9). Leaving out each patient changed spot-level d by ≤13.9% and hierarchical M0 coefficients by ≤10.9%, but section-paired d_z by up to 64.7% (MYC_TF_clean, patient 22). MYC belongs to the Silencing signature.

In the pseudo-spot stress test with composition differences and no introduced expression shift, the unweighted mean proportion of M3 95% intervals containing zero across the four stromal genes was 85.0% (Monte Carlo interval 79.5–90.2%), below the prespecified 90% criterion. Under source-cell-type expression shifts, with E-like and D-like compositions sampled from the same within-section pool, all 333 accepted primary-gene M3 fits on the normalized scale contained the defined average effect on E-like pseudo-spots (Supplementary Figure S5; Supplementary Table S10). The gene–drug audit retained 182 DGIdb records with a recorded interaction type across 20 of 48 queried genes (135 E-assigned, 47 D-assigned). PDGFRB contributed 85: 17 with recorded approval status (13 active principles after mechanical salt consolidation) and 68 retained only through an eight-gene whitelist. These annotations do not indicate efficacy or region-specific response (Supplementary Table S11).

## 4. Discussion

E-labeled regions combined higher regional matrix-associated expression with greater estimated cancer-associated fibroblast (CAF) abundance than D-labeled regions. In the secondary dataset, pathology annotations showed a lower tumor-annotated fraction in E-labeled regions of the same capture, and in discovery, ambiguous spots were enriched in mixed D/E neighborhoods. We call this correspondence a stromal–transcriptomic contrast: a descriptive link among transcriptomic labels, regional stromal and matrix-associated expression, and, where annotations existed, tumor–stroma composition. Expression, composition, and spatial organization are related measurements that share data and biological context. Cohorts and units of analysis are summarized in Table 1 and Supplementary Figure S1.

The D, E, and I labels borrow the vocabulary of inflamed, excluded, and desert phenotypes but are defined differently. Keren et al. [3] classified TNBCs as cold, mixed, or compartmentalized from tumor–immune cell relationships in multiplexed images, whereas Gruosso et al. [5] and Hammerl et al. [4] used CD8-positive T-cell localization relative to tumor, stromal, and border compartments. These are tumor-level categories based on identified cells, whereas our labels were assigned to multicellular spots from transcriptomic scores and percentile gates, and one section could contain D-, E-, and I-labeled regions; they describe regional expression states (Supplementary Table S1). The reference E−D contrast of the 770-configuration threshold sweep kept its direction in every configuration (Supplementary Figure S2; Supplementary Table S3), and the CAF-abundance and PDGFRB contrasts kept their sign across the twelve-configuration labeling battery (Supplementary Table S4); that battery is not reported for the collagen genes. Removing half the classifier genes lowered label agreement to 65.2% but retained 0.84 of the spot-level effect. Unsupervised clustering agreed little with the labels on globally scaled data and followed patients, reaching the prespecified signal criterion only after within-section scaling or spatial smoothing with Harmony [29] (Supplementary Figure S3; Supplementary Table S3); agreement therefore depended on preprocessing.

The clearest molecular component was regional. E-labeled regions had higher CAF abundance and higher PDGFRB, COL3A1, COL5A1, and COL6A1 expression than D-labeled regions, and after adjustment for sequencing depth and estimated tumor abundance, the three collagen genes, but not clearly PDGFRB, also exceeded I-labeled regions (Figure 1A,B; Supplementary Table S4). This fits the heterogeneity of breast-cancer stroma: Costa et al. [7] linked CAF-S1 to an immunosuppressive environment, S. Z. Wu et al. [9] resolved myofibroblast-like and inflammatory-like stromal states in TNBC, and Kieffer et al. [8] divided CAF-S1 into transcriptionally distinct clusters in breast cancer, with immunotherapy-resistance associations that included melanoma and non-small-cell lung cancer. Because CAF phenotypes are context-dependent [10], the collagen pattern is not assigned to CAF-S1 or any published taxonomy. RNA abundance does not measure collagen deposition or tissue mechanics, so the pattern supports matrix-associated regional differentiation.

Cell-type-conditioned analyses refined this picture. Within CAFs, C-SIDE estimated higher COL3A1 and COL5A1 in E-labeled spots in all four arms, PDGFRB depended on the source of cell-type weights (Holm-supported under A0 but not RCTD weights), and COL6A1 coefficients were negative despite its positive regional contrast (Figure 1C; Supplementary Table S5). Compatible explanations for the COL6A1 divergence are composition (more CAFs or other COL6A1-expressing cells in E-labeled spots), CAF state differences between regions, and dependence on reference profiles and model specification. State-dependent CAF variation has precedent: some RNA-defined CAF phenotypes can be recovered at the protein level, with modality-specific classification boundaries [30], and immunosuppressive fibroblast programs show spatial plasticity in breast cancer [31], although neither study corresponds to the present labels. C-SIDE estimates expression conditional on reference-derived weights [16], and the four arms reuse overlapping spots in a pooled model. The attribution to CAFs is therefore selective by gene rather than uniform.

Pathology annotations addressed a related but distinct question. Within the 11 cancer captures of the secondary dataset, tumor-annotated spots formed a smaller fraction of tumor- plus stroma-annotated spots in E- than in D-labeled regions of the same capture (median difference −0.141; negative in nine captures and tied in two saturated captures; Figure 2A,B; Table 2). Restricting stroma to tumor-associated stroma and fibrosis preserved the median, whereas removing in situ annotations increased the ties from two to four and brought the upper bound of the interval to zero (Supplementary Table S6); the correspondence was therefore informative but sensitive to the definition of tumor. Lower tumor and higher stromal fractions are complementary expressions of one contrast in annotated-spot composition. The secondary dataset comprised 11 triple-negative tumors, including three claudin-low and four metaplastic tumors [21], so the finding is interpreted within that dataset. The discovery sections were originally used to describe a conserved spatial architecture with a stromal–immune cluster dispersed from tumor clusters [17]; because we reanalyzed them, this agreement is contextual.

Mixed-label adjacency and annotation-defined interface proximity captured different spatial properties. In discovery, Ambiguous_Cold spots were enriched for neighborhoods containing both D- and E-labeled spots relative to both spatial null models (0.401 versus null intervals of 0.302–0.369 and 0.226–0.286), and in 36 of 43 sections their group means for at least four of six measurements lay between the D and E interior means (Figure 2C; Supplementary Table S7). In the secondary cohort, proximity to the annotated tumor–stroma interface did not clearly separate Ambiguous_Cold from pooled D/E spots (median AUC 0.486, bootstrap interval 0.411–0.554; 7 of 11 cancer captures evaluable; Figure 2D). An interval that includes 0.5 leaves the relationship uncertain rather than excluding it. The two analyses address adjacency among transcriptomic labels and distance to a pathology-derived boundary, respectively, which parallels the observation that cellular abundance, state, proximity, and higher-order organization can carry different information about immunotherapy response in TNBC [6].

Regional immune differences were smaller and more dependent on analytical definition than the stromal contrasts. With the score_genes constructor, seven of eight readouts passed Holm correction before adjustment; after adjustment for 15 estimated cell-type abundances, all estimates decreased and four remained Holm-significant, with concordant attenuation for seven of eight readouts across four deconvolution specifications (Figure 3A,B; Supplementary Table S8). Holm decisions varied with parametrization: three readouts remained significant with a reduced covariate set and none with principal components, although directions were retained. Panel comparisons D1–D4 were Holm-significant in all four comparisons before adjustment but in two after it; because the compared panels differ in gene content and in the sampled control-gene background, the differences cannot be attributed to individual genes. Mean-expression constructors supported fewer contrasts, and control-gene means tracked sequencing depth. Library size is itself related to tissue structure in spatial data [32], so adjustment for sequencing depth can remove biological as well as technical variation. Section-level meta-analysis gave positive mean differences for all readouts, but with substantial heterogeneity and prediction intervals that included zero (Figure 3C). The 11 secondary cancer captures had the discovery sign for every mean-constructor readout, but none passed Holm correction, which supports directional corroboration rather than validation (Figure 3D); only HLA-A+B2M passed in the 15-capture sensitivity set.

MYC mRNA and two MYC-associated expression scores were higher in D- than in I-labeled regions (Supplementary Figure S4; Supplementary Table S9). Adjustment for estimated tumor abundance, with which the two scores correlated, reduced Hallmark36 (0.417 to 0.155) and MYC_TF_clean (0.486 to 0.179) far more than MYC mRNA (0.330 to 0.285), and separate adjustment for log UMI showed the same pattern. MYC has been linked to tumor immune evasion, including regulation of CD47 and PD-L1 [33,34]. Experimental studies in TNBC models make an immune-modulating role for MYC plausible through different routes: DNMT1 upregulation with suppression of cGAS–STING signaling [35] and MIZ1-dependent repression of interferon signaling [36]. Because MYC was compared between D and I regions, the stromal and immune contrasts compared E with D, and no perturbation was performed, these mechanisms remain context for a regional association. In the pseudo-spot stress test with composition differences and no introduced shift, 85.0% of normalized-scale M3 intervals contained zero, below the prespecified 90% criterion (Supplementary Figure S5; Supplementary Table S10); this proportion is not a type I error rate or the coverage of the true M3 coefficient. In the shift scenario, all 333 accepted intervals contained the defined average effect on E-like pseudo-spots, an estimand not necessarily identical to the adjusted regression coefficient. Adjusted residual contrasts should therefore not be read as cell-intrinsic effects.

Pharmacological annotation drew on DGIdb, which consolidates drug–gene interaction claims [28]; the 182 prioritized records covered 20 of 48 queried genes (Supplementary Table S11). Of the 85 prioritized PDGFRB records, 68 were retained only through the eight-gene whitelist, showing how the prioritization rules shaped its annotation count. Recorded approval status describes the compound, not approval in TNBC, and neither the annotations nor the regional PDGFRB association indicate that E-labeled regions depend on PDGFRB signaling or would respond to a recorded inhibitor.

Analyses distinguished patient, section, capture, spot, and pooled cell-type units, controlled multiplicity within defined families, and examined model, label, constructor, deconvolution, and influence sensitivities (Supplementary Table S2). Material limitations remain: the labels were built from expression programs related to some downstream readouts (a circularity comparable to double dipping in clustering-based analyses [37,38]); spots contain several cells; deconvolution and cell-type attribution depend on reference profiles and model specification; several resampling analyses held labels, scores, or weights fixed; and the hierarchical models, meta-analysis, pathology comparisons, and pooled C-SIDE analyses used different units and dependence assumptions, so their agreement is not replicable. The secondary cohort was small and heterogeneous, and the stress test used the true composition of each pseudo-spot whereas real data used estimated composition. Within these boundaries, the study shows how a matrix-associated regional signal, annotated tumor–stroma composition, mixed-label neighborhoods, and cell-type-conditioned estimates relate and where they diverge. These findings support an estimand-aware interpretation of spatial TNBC data, in which regional expression, annotated composition, neighborhood organization, and cell-type-conditioned estimates provide complementary information.

## 5. Conclusions

Among transcriptionally defined cold regions of triple-negative breast cancer (TNBC), E-labeled regions had higher estimated cancer-associated fibroblast (CAF) abundance and matrix-associated expression than D-labeled regions; three collagen genes also remained higher in E-labeled than in D- and I-labeled regions after adjustment for sequencing depth and estimated tumor abundance. We refer to this relation among operational labels, regional stromal expression, and complementary compositional measurements as a stromal–transcriptomic contrast. In the secondary dataset, E-labeled regions had a lower tumor-annotated fraction than D-labeled regions within captures, a finding sensitive to the anatomical definition of tumor; in discovery, Ambiguous_Cold spots were enriched in mixed D/E neighborhoods under two spatial null models. Cell-type-conditioned estimates were concordant for *COL3A1* and *COL5A1* but opposite for *COL6A1*, so the regional collagen pattern is not a uniform increase within CAFs, and immune readouts depended on panel, constructor, and compositional adjustment. Spatially registered pathology and cell-resolved measurements with patient-level replication could distinguish compositional differences from expression changes within defined populations. Together, these findings support interpreting spatial TNBC heterogeneity through complementary measures of regional expression, composition, and local organization.

## Figures and Tables

**Figure 1:**
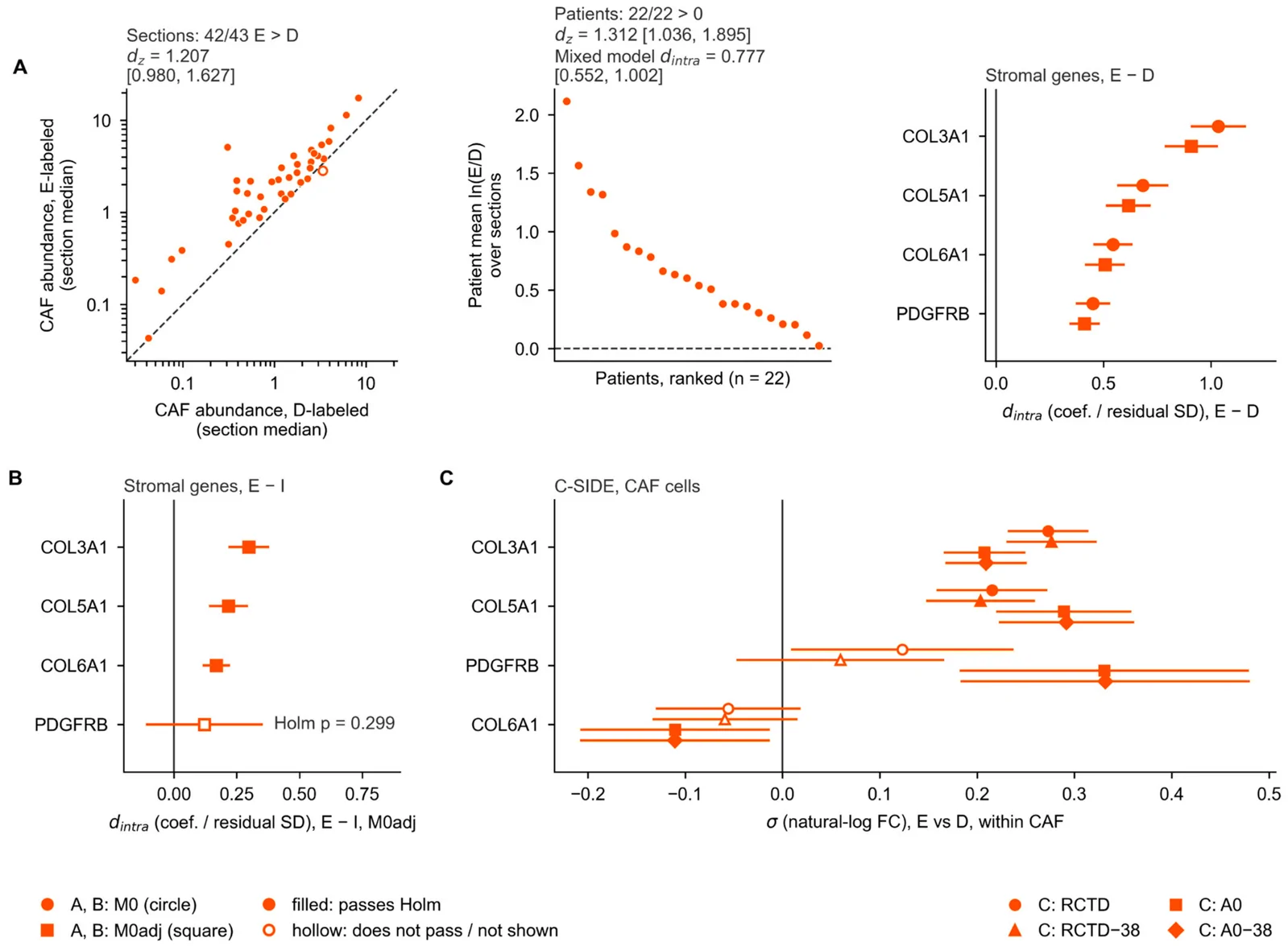
Regional stromal contrasts and CAF-conditioned expression estimates. Discovery dataset (GSE210616; 43 sections, 22 patients; 14,884 E- plus D-labeled spots). (**A**) E−D standardized within-section differences (d_intra, the group coefficient divided by the residual SD of the same hierarchical fit; 95% confidence intervals) for estimated CAF abundance under M0 and for PDGFRB, COL3A1, COL5A1 and COL6A1 under M0 and M0adj (adjusted for log UMI and estimated tumor abundance). (**B**) E−I differences for the same four genes under M0 and M0adj (5582 E- and 4080 I-labeled spots). Filled symbols indicate Holm-adjusted *p* < 0.05 within the corresponding family: CAF plus four genes under M0 in A (m = 5); four-gene families for M0adj in A and for both models in B (m = 4). (**C**) C-SIDE coefficients within CAFs (σ, natural-log fold change, E versus D) for the four genes in four pooled arms using RCTD or A0 weights, with or without Section 38 [13] (10,842–14,602 modeled spots per arm). Filled symbols indicate Holm-adjusted *p* < 0.05 computed on original *p*-values among estimable gene–cell-type pairs. The arms are related sensitivity analyses on overlapping data. Numerical values are given in Supplementary Tables S4 and S5.

**Figure 2:**
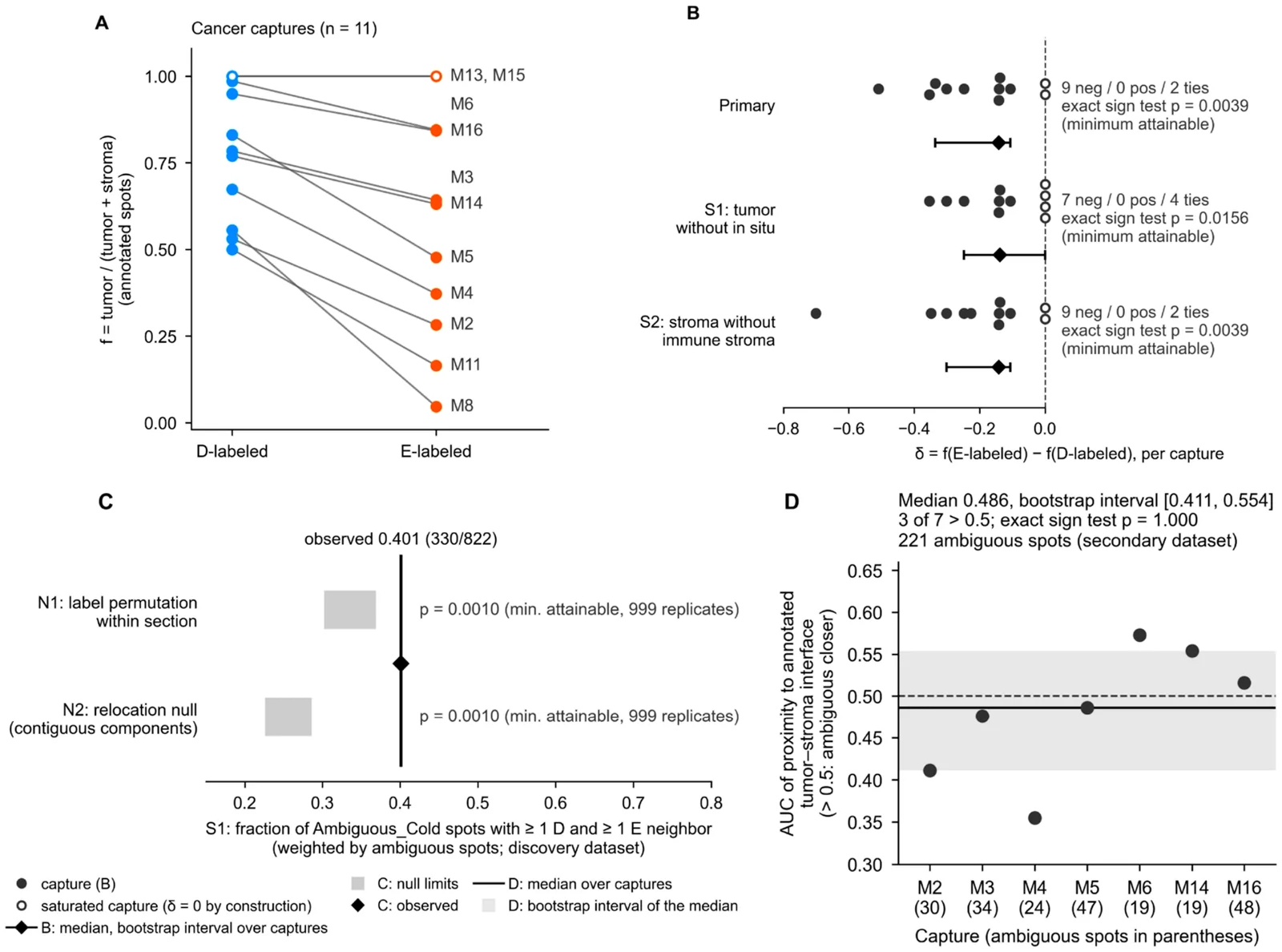
Annotated composition and mixed neighborhoods. (**A**,**B**) Secondary dataset (GSE213688), 11 cancer captures, one per donor label. (**A**) Fraction of tumor-annotated spots among tumor- plus stroma-annotated spots in D- and E-labeled regions, paired within capture. (**B**) Within-capture difference δ = f(E) − f(D), with the median (−0.141) and 95% capture-bootstrap interval (−0.335 to −0.107; 10,000 draws); the two saturated captures (M13, M15; δ = 0) are shown. Exact counts are given in Table 2. (**C**) Discovery dataset (GSE210616; 43 sections): ambiguous-spot-weighted fraction of Ambiguous_Cold spots (*n* = 822) with at least one D- and one E-labeled spot among their six nearest Euclidean neighbors (0.401), compared with reported null intervals from within-section label permutation (N1) and rigid relocation of contiguous ambiguous components onto existing spot sites (N2; 999 replicates each). (**D**) Secondary dataset: per-donor AUC for hexagonal-grid proximity of Ambiguous_Cold versus pooled D/E spots to the annotated tumor–stroma interface (7 of 11 cancer captures evaluable, one capture per donor; 221 Ambiguous_Cold spots); dashed line, AUC = 0.5. Operational labels were retained without recalculating thresholds after restriction to cancer captures. Numerical values are given in Table 2 and Supplementary Tables S6 and S7.

**Figure 3:**
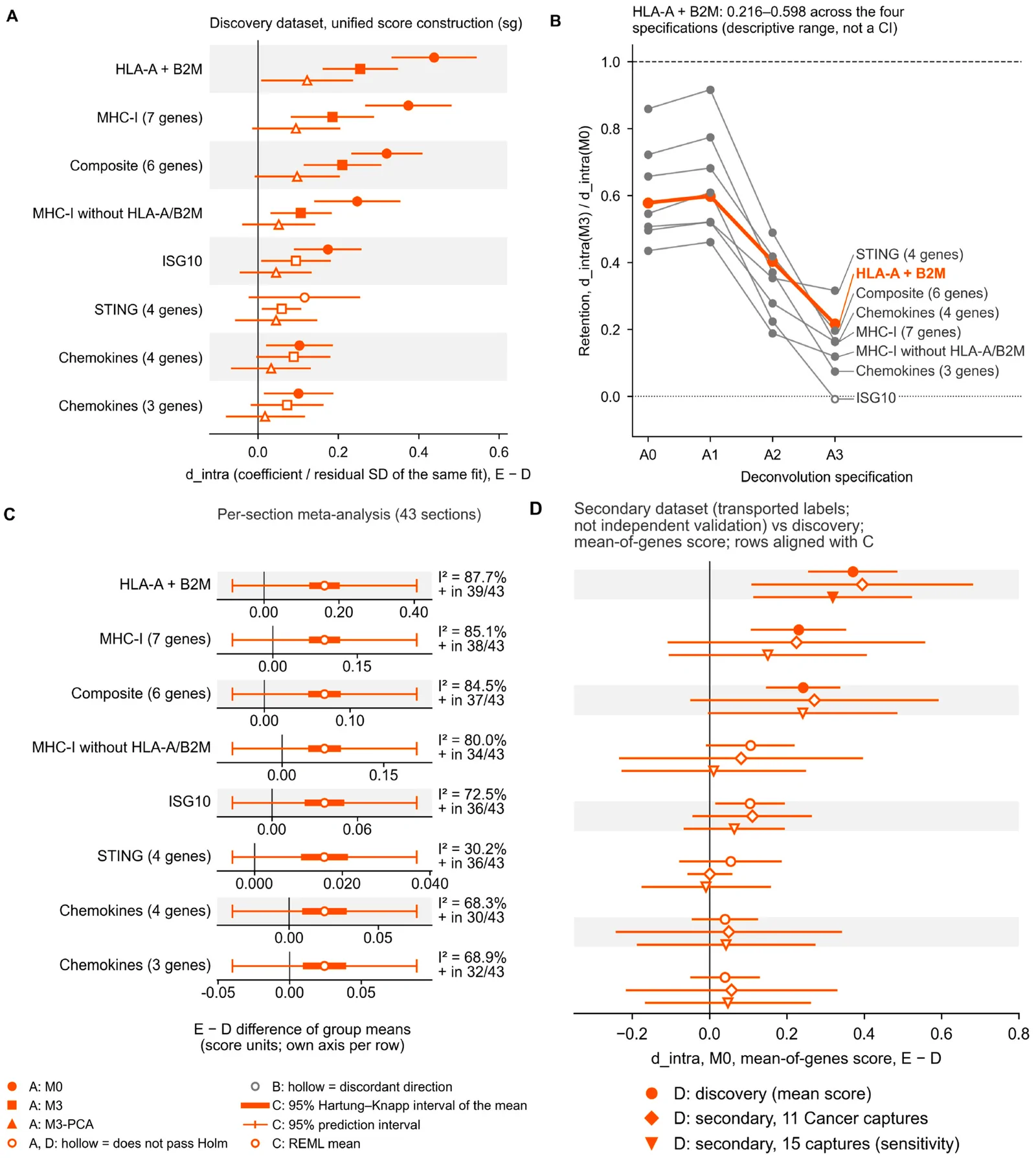
Regional immune readouts, E-labeled versus D-labeled regions. (**A**–**C**) Discovery dataset (43 sections, 22 patients). (**A**) d_intra (95% confidence intervals) for eight prespecified readouts constructed with score_genes under M0 (no covariates) and M3 (15 estimated cell-type abundances); filled symbols indicate Holm-adjusted *p* < 0.05 (m = 8 per model). (**B**) Ratio of M3 to M0 standardized effects for each readout under four deconvolution specifications (A0–A2, Cell2location; A3, Stereoscope). Each effect is standardized by the residual SD of its own model. (**C**) Section-level random-effects meta-analysis (REML with Hartung–Knapp adjustment; spatial-block bootstrap standard errors): mean E−D difference in score units, with 95% confidence interval and 95% prediction interval; scales differ between readouts and from panels A and D. (**D**) M0 d_intra with the mean log-normalized constructor in discovery (43 sections) and in 11 secondary cancer captures (capture as unit); filled symbols indicate Holm-adjusted *p* < 0.05. Operational labels were retained without recalculating thresholds after restriction to cancer captures. Numerical values are given in Supplementary Table S8.

**Table 1:** Cohorts, analytic populations, and units of analysis.

Cohort	Analytic Role	Object and Population	Unit(s) of Analysis
GSE210616—Visium	Discovery; operational classification, regional contrasts, and spatial analyses.	22 patients, 43 sections, and 49,464 spots. Label distribution: D, 9302; E, 5582; I, 4080; Ambiguous_Cold, 822; Normal_Stroma, 29,678. E+D subset: 14,884 spots. Cold subset (D+E+Ambiguous_Cold): 15,706 spots.	Regional models: patient → section → spot, with specification B as primary and specification A as a sensitivity analysis. Meta-analysis: section. C-SIDE: pooled spots, with 14,715–14,884 input spots and 10,842–14,602 modeled spots, depending on the arm.
GSE213688—Visium	Secondary cohort for directional corroboration; annotated pathological composition and proximity to the tumor–stroma interface.	15 captures, 25,907 spots, and 12 donor labels. The 11 cancer captures carry 11 labels; the four Normal captures carry two, one of which is shared with a cancer capture. Primary population: 11 cancer captures, with 6254 E+D spots (E, 2410; D, 3844). Sensitivity: all 15 captures, with 7612 E+D spots (E, 2952; D, 4660).	Regional models: capture → spot, specification A. Annotated composition, P9: capture, with 11 evaluable cancer captures. Interface proximity: donor, with seven evaluable captures corresponding to seven donor labels.
GSE176078—scRNA-seq	Reference for deconvolution and cellular source for the pseudo-spot stress test.	100,064 loaded cells and 98,418 retained cells, distributed across 15 cell types or states. TNBC subset for simulation, partitioned by patient: Ref, five patients and 22,966 cells; Eval, five patients and 19,546 cells.	Deconvolution: cell-type expression profiles. Simulation: replicate with 14,884 pseudo-spots distributed across 43 pseudo-sections.

Footnote: D, Desert; E, Excluded; I, Inflamed; E+D, spots labeled Excluded or Desert; TNBC, triple-negative breast cancer; scRNA-seq, single-cell RNA sequencing; Ref/Eval, patient-level partition into reference and evaluation subsets. Labels represent operational transcriptomic states; Normal_Stroma is a computational identifier. In GSE213688, donor labels were derived from GEO sample titles, and transcriptomic labels were retained without recalculating thresholds after restricting analyses to cancer captures. Spot counts represent observational sizes, not numbers of independent biological replicates. Model specifications and eligibility criteria are detailed in Supplementary Table S2.

**Table 2:** Annotated tumor–stroma composition by cancer capture in the secondary dataset (primary dictionary). Counts are tumor-annotated/stroma-annotated spots within each operational label; other annotation categories are excluded from the denominator. f = tumor/(tumor + stroma); δ = f(E) − f(D). Tumor = tumor + uncertain tumor + in situ; stroma = tumor-associated stroma + fibrosis + immune stroma. Saturated = evaluable capture with no stroma-annotated spots in either label (δ = 0 by construction).

Capture	Donor label	Subtype	D: Tumor/ Stroma Spots	E: Tumor/ Stroma Spots	f(D)	f(E)	δ = f(E) − f(D)	Status
M2	CL-like1	CL-like	97/86	51/130	0.530	0.282	−0.248	Evaluable
M3	Unstable1	Unstable	286/79	149/83	0.784	0.642	−0.141	Evaluable
M4	Unstable2	Unstable	70/34	26/44	0.673	0.371	−0.302	Evaluable
M5	Unstable3	Unstable	332/68	71/78	0.830	0.477	−0.353	Evaluable
M6	Unstable4	Unstable	338/5	49/9	0.985	0.845	−0.141	Evaluable
M8	CL-like2	CL-like	45/36	2/41	0.556	0.047	−0.509	Evaluable
M11	CL-like3	CL-like	90/90	13/66	0.500	0.165	−0.335	Evaluable
M13	MBC_spindle1	Metaplastic	119/0	152/0	1.000	1.000	0.000	Evaluable, saturated
M14	MBC_spindle2	Metaplastic	80/24	53/31	0.769	0.631	−0.138	Evaluable
M15	MBC_chondroid	Metaplastic	246/0	275/0	1.000	1.000	0.000	Evaluable, saturated
M16	MBC_NST	Metaplastic	277/15	144/27	0.949	0.842	−0.107	Evaluable

Footnote: Across 11 captures: median δ −0.141 (95% capture-bootstrap interval −0.335 to −0.107); 9 negative, 0 positive, 2 ties; two-sided sign test excluding ties, *p* = 0.0039. The primary-dictionary fractions were based on 2417 D-labeled and 1494 E-labeled tumor-or-stroma-annotated spots. Values are proportions of annotated spots, not of cells or tissue area. Dictionary variants and the non-saturated sensitivity are reported in Supplementary Table S6.

## Data Availability

The spatial transcriptomics datasets analyzed in this study are publicly available in the NCBI Gene Expression Omnibus (GEO) under accession numbers GSE210616 (discovery dataset) and GSE213688 (secondary dataset used for directional corroboration). The single-cell RNA-sequencing dataset used as the reference for cell-type deconvolution and as the source of cells for the pseudo-spot stress test is available under accession number GSE176078. These public data, the AnnData (.h5ad) objects derived from them and the model weights are not redistributed. Drug–gene annotation used existing records from the Drug–Gene Interaction Database (DGIdb) [28], queried on 20 August 2026; the database release was not recorded, and the records were audited without new queries (Section 2.7). The analysis code, the results of each analysis step, the source data and scripts for the figures, Supplementary Tables S3–S11 with a Supplementary Information file, documentation (including the package versions of the three computational environments) and a file manifest with SHA-256 checksums are deposited in Zenodo at https://doi.org/10.5281/zenodo.23487654 under the MIT license. Environment export files (Conda YAML or lock files) were not archived. The deposit does not contain the training code of the primary abundance specification (A0); the scripts that built the intermediate analysis objects come from an earlier version of the pipeline and are provided for reference. Supplementary Tables S1–S11 give the operational classification rules, model specifications, and numerical estimates.

## Supplementary Materials

The following supporting information can be downloaded at: https://www.mdpi.com/article/10.3390/cancers18213476/s1, Figure S1: Analytical structure and data flow of the TNBC spatial-transcriptomic reanalysis; Figure S2: Sensitivity of operational labels and regional contrasts; Figure S3: Unsupervised clustering of cold spots; Figure S4: MYC measures, D-labeled versus I-labeled regions; Figure S5: Pseudo-spot stress test of the composition-adjusted model (M3); Table S1: Operational Classification Rules and Gene-Expression Readouts; Table S2: Model Specifications, Inferential Procedures, and Sensitivity Analyses; Table S3: Operational-label and regional-contrast sensitivity; Table S4: Regional stromal and matrix-associated contrasts; Table S5: Selected cell-type-conditioned C-SIDE estimates; Table S6: Sensitivity of annotated tumor–stroma composition; Table S7: Mixed-label neighborhoods and annotated-interface proximity; Table S8: Regional immune readouts and key sensitivity analyses; Table S9: MYC-associated measurements and adjustment sensitivity; Table S10: Summary of the pseudo-spot stress test; Table S11: Prioritized drug–gene annotations by gene.

## Abbreviations

The following abbreviations are used in this manuscript:

**Abbreviation**	**Definition**
ARI	Adjusted Rand Index
ATT	Average Treatment Effect on the Treated
AUC	Area Under the Receiver Operating Characteristic Curve
BH	Benjamini–Hochberg procedure
CAF	Cancer-Associated Fibroblast
C-SIDE	Cell Type-Specific Inference of Differential Expression
DGIdb	Drug–Gene Interaction Database
GEO	Gene Expression Omnibus
ISG	Interferon-Stimulated Gene
MHC-I	Major Histocompatibility Complex Class I
PCA	Principal Component Analysis
PD-1	Programmed Cell Death Protein 1
RCTD	Robust Cell Type Decomposition
REML	Restricted Maximum Likelihood
scRNA-seq	Single-Cell RNA Sequencing
SD	Standard Deviation
SE	Standard Error
TGFβ	Transforming Growth Factor Beta
TNBC	Triple-Negative Breast Cancer
UMI	Unique Molecular Identifier
