# Supplementary material for: Matrix-Associated Gene Expression and Annotated Tumor–Stroma Composition of Transcriptomically Defined Cold Regions in Triple-Negative Breast Cancer
Source: Cancers (Basel). 2026 Oct 28;18(21):3476. doi: 10.3390/cancers18213476 (PMC13761535; doi:10.3390/cancers18213476)

SUPPLEMENTARY INFORMATION

Supplementary Table S1. Operational Classification Rules and Gene-Expression Readouts

Block A. Signatures and Sequential Classification

Component	Genes or input	Transformation or decision rule	Output and methodological qualification
**Tumor signature** (5 genes)	EPCAM, KRT8, KRT18, KRT19, MKI67	Signature expression score.	Input to the Tumor gate; nominal gene list.
**CD8 signature (7 genes)**	CD8A, CD8B, CD3D, CD3E, GZMA, GZMB, PRF1	Signature expression score.	Input to the immune gate; nominal gene list.
**Silencing signature (7 genes)**	MYC, EZH2, SUZ12, CTNNB1, ATF3, STAT3, DNMT1	Signature expression score.	Positive component of $Silencing_{norm} - Barrier_{norm}$
**Barrier signature (9 genes)**	COL1A1, COL1A2, COL10A1, FN1, POSTN, TGFB1, ACTA2, FAP, THBS2	Signature expression score.	Subtracted component of $Silencing_{norm} - Barrier_{norm}$; "Barrier" is a computational identifier.
**Scoring and standardization**	Four signatures comprising 28 unique genes.	Scanpy score_genes, followed by within-sample_id z-standardization (ddof = 0); groups with near-zero standard deviation and non-finite standardized values are assigned zero.	Classification uses the standardized score columns stored in the analysis object; their complete generation provenance is unavailable.
**Gate 1: Normal_Stroma**	Standardized Tumor score T; t_{60}, its 60th percentile across all spots in the corresponding object.	Assign Normal_Stroma if $T < t_{60}$.	Spots equal to t_{60} proceed to Gate 2. A universal numerical cutoff across cohorts is not assumed.
Gate 2: **Inflamed (I)**	Standardized CD8 score C; c_{75}, its 75th percentile across all spots, not only those passing the Tumor gate.	Among spots not assigned Normal_Stroma, assign I if $C > c_{75}$.	Spots equal to c_{75} proceed to Gate 3.
**Gate 3: D, E, or Ambiguous_Cold**	$M = Silencing_{norm} - Barrier_{norm}$	Among remaining spots: $M > 0.10$, assign D; $M < -0.10$, assign E; $-0.10 \leq M \leq 0.10$, assign Ambiguous_Cold	Both ambiguity boundaries are included. D and E denote local transcriptomic states, not histological compartments.
**Secondary-cohort handling**	Labels in the GSE213688 classified object.	Retain existing labels after restriction to Cancer captures; thresholds are not recalculated for this restriction.	Retaining labels does not establish transfer of identical numerical cutoffs or identical effective gene sets between cohorts.

Block B. Regional Expression Readouts

Family and readout	Genes or measurement	Nominal/ effective size	Constructor and primary contrast	Methodological qualification
A: STING	TMEM173, TBK1, IRF3, CGAS	4/4	Discovery: SG and mean; secondary: mean. E–D.	Expression summary, not a measure of pathway activation.
**A: ISG10**	IFIT1, IFIT2, IFIT3, MX1, OAS1, OAS2, OASL, ISG15, RSAD2, IFI44L	10/10	Discovery: SG and mean; secondary: mean. E–D.	An alternative sensitivity panel replaces OASL with MX2 and uses mean log-expression.

**A: HLA-A + B2M**	HLA-A, B2M	2/2	Discovery: SG and mean; secondary: mean. E–D.	Shares genes with the MHC-I panels and the six-gene composite.
**A: Seven-gene MHC-I panel**	HLA-A, HLA-B, HLA-C, B2M, TAP1, TAP2, TAPBP	7/7	Discovery: SG and mean; secondary: mean. E–D.	Overlaps other immune panels; panels are not independent measurements.
**A: MHC-I without HLA-A/B2M**	HLA-B, HLA-C, TAP1, TAP2, TAPBP	5/5	Discovery: SG and mean; secondary: mean. E–D.	Evaluates sensitivity to panel composition.
**A: Six-gene immune composite**	CXCL9, CXCL10, CXCL11, CCL5, HLA-A, B2M	6/6	Discovery: SG and mean; secondary: mean. E–D.	Contains four chemokines and two antigen-presentation-related genes; not a six-chemokine panel.
**A: Four-chemokine panel**	CXCL9, CXCL10, CXCL11, CCL5	4/4	Discovery: SG and mean; secondary: mean. E–D.	Overlaps the three-chemokine panel and the six-gene composite.
**A: Three-chemokine panel**	CCL5, CXCL9, CXCL10	3/3	Discovery: SG and mean; secondary: mean. E–D.	One of the eight Family A readouts.
**B: Estimated CAF abundance**	Posterior mean CAF abundance from primary deconvolution specification A0.	Not applicable	Deconvolution estimate. E–D.	Model-based abundance, not an observed histological cell count or measured physical density.
**B: Individual stromal genes**	PDGFRB; COL3A1; COL5A1; COL6A1	1/1 per gene	Individual log-normalized expression. E–D and E–I.	Four separate readouts outside the classifier signatures; absence of direct overlap does not imply statistical independence.
**C: MYC mRNA**	MYC	1/1	Individual log-normalized expression. D–I.	Direct classifier overlap: MYC belongs to the Silencing signature.
**C: Hallmark36**	Author-defined MYC-target panel: AP5Z1, BRPF3, CCT6P3, CDK4, CHD7, CNBP, CSDE1, DNMT3B, EIF2B5, FADS3, FBXL14, GANAB, GNL3, GPT2, HSPE1, INTS1, KIF20B, LARP1, MCM4, MDM2, MECP2, METAP2, MRE11, MYB, NME1, NOL5A, NPM1, POLD2, POLR1B, RBMX, RPS13, RRM1, SEC63, SLC37A4, SLMO2, STARD10.	36 nominal/33 measurable	MYC-target expression score. D–I.	CCT6P3, NOL5A, and SLMO2 are absent or constant. The panel is not the complete MSigDB Hallmark collection; see note e.
**C: MYC_TF_clean**	Expression-derived MYC target score available in the analysis object.	Not established for the analyzed column	Available score analyzed as supplied. D–I.	Not a direct measurement of TF activity. The exact target set and removal of classifier overlap were not established for this column.

Notes to Supplementary Table S1:

a. Signature sizes in Block A are nominal and should not be interpreted as verified effective sizes in every cohort. The classification code invokes score_genes without explicitly setting ctrl_size, n_bins, or random_state; these parameters therefore follow the installed version’s defaults. The documented Scanpy 1.9.x defaults are ctrl_size=50, n_bins=25, and random_state=0. Classification-score generation is distinguished from the primary readout and control-gene analyses.

b. SG denotes score_genes applied to discovery log-normalized .raw expression. The parallel discovery constructor is mean log-expression over the effective gene list. Secondary immune readouts use mean expression from the log-normalized preprocessed .X matrix. Primary readout analyses use seed 0, ctrl_size=50, and n_bins=25; seed-sensitivity analyses use 20 seeds.

c. Immune-panel sizes in Block B refer to regional analyses, not to gene coverage after C-SIDE filtering. The alternative ISG panel replaces OASL with MX2 and is evaluated separately from the primary eight-readout Holm family.

d. The numerical Tumor cutoff of 0.24660672 applies to the documented GSE213688 classification and is not assigned to discovery. Retaining secondary labels after restriction to Cancer captures is distinct from demonstrating transfer of numerical thresholds between cohorts.

e. Hallmark36 is an author-defined panel recorded as a 36-of-172-gene MSigDB-derived selection. The listed definition is consistent with the reported nominal size and three absent or constant genes; its identity with the exact list used to generate the analyzed score was not independently verified. For MYC_TF_clean, the available documentation does not establish the complete generation procedure, target count, or classifier-overlap removal for the analyzed column. These properties are not inferred from the score name.

f. D, Desert; E, Excluded; I, Inflamed; CAF, cancer-associated fibroblast; TF, transcription factor; SG, score_genes. Contrasts are expressed as the first label minus the second. Removing directly overlapping genes does not remove dependence between correlated expression programs. “Barrier” and “Normal_Stroma” are computational identifiers, not validated anatomical categories.

Supplementary Table S2. Model Specifications, Inferential Procedures, and Sensitivity Analyses

Block A. Regional Models and Grouping Specifications

Model or specification	Covariates and transformations	Grouping structure	Estimand or application	Methodological qualification
M0	Group indicator and section mean of group; no abundance or depth covariates.	Specification B in discovery; specification A in the secondary cohort.	Group coefficient β; $d_{intra} = \beta / SD_{residual}$ from the same fit.	“Unadjusted” denotes absence of abundance or depth adjustment; grouping and Mundlak terms remain.
**M1**	M0 plus estimated Tumor abundance, centered within section, and its section mean.	Same grouping as the corresponding M0 analysis.	Tumor-conditioned regional contrast.	Estimated abundance is not a measured tumor fraction.
**M2**	M1 plus estimated CAF abundance, centered within section, and its section mean.	Same grouping as the corresponding M0 analysis.	Tumor- and CAF-conditioned contrast.	Statistical conditioning does not establish causal mediation.
**M3**	M0 plus 15 estimated cell-type abundances, centered within section, and their section means.	Same grouping as the corresponding M0 analysis.	Composition-conditioned regional contrast.	Composition is estimated in real data. For A3, Tumor is omitted because the scaled proportions sum to a constant.
**M3-PCA**	M0 plus three principal components derived from log1p-transformed, standardized abundance estimates, with within-section and section-mean terms.	Same grouping as the corresponding M0 analysis.	Contrast conditioned on a reduced representation of abundance.	Alternative parameterization; it does not establish elimination of collinearity or confounding.
**M3-RED**	M0 plus Tumor, CAF, total T cells, NK + NKT, and Endothelial, with within-section and section-mean terms.	Same grouping as the corresponding M0 analysis.	Contrast conditioned on five aggregated groups.	Total T cells = CD4_T + CD8_T + T_Cell_Cycling.
**M0adj: stromal adjustment**	M0 plus $\log(UMI)$ and estimated Tumor abundance, with corresponding section-mean terms.	Same grouping as the corresponding regional analysis.	Adjusted E−D and E−I stromal-gene contrasts.	Also used in the stromal section bootstrap.

**Depth-adjusted variants**	M0 plus log (UMI), or M1 plus log (UMI), as specified for each analysis.	Same grouping as the corresponding baseline analysis.	Sensitivity to library depth and differences between score constructors.	Each model is defined by its actual covariates; similarly named variants are not assumed to be interchangeable.
**Grouping specification A**	Fixed-effect structure of the corresponding model.	Capture/section random intercept and group slope, with a 2 x 2 covariance matrix; no patient level.	Primary grouping in the secondary cohort; grouping sensitivity in discovery.	Omission of a patient level does not demonstrate absence of within-patient dependence.

Block B. Inferential and Sensitivity Procedures

Procedure	Estimand or methodological question	Analytical or resampling unit	Design	Inference and methodological qualification
Hierarchical spatial bootstrap	Uncertainty in regional group coefficients.	Patient → section within sampled patient → spatial block within sampled section; sampling with replacement at each level.	500 paired draws, seed 42, shared across readouts and variants. Recursive-quadrant blocks split at bounding-box midpoints, stopping at ≤40 spots or depth 3. Mixed models are refitted; repeated patient/section copies receive unique identifiers, and section means are recalculated within copies.	Percentile 95% coefficient intervals and a tail summary relative to zero without an imposed null. Readout–model cells with >30% failed attempted draws are non-interpretable under an operational rule.
**Stromal section bootstrap**	Uncertainty in depth- and Tumor-adjusted stromal contrasts.	Section, sampled with replacement; not patient.	1,000 draws; M0adj; E−D and E−I.	Coefficient intervals use accepted draws; attempted and accepted counts are distinguished. Patient-level resampling is not implied.
**Section-level meta-analysis**	Mean E−D difference and between-section heterogeneity.	Section; at least 10 spots per group.	Spatial-block bootstrap standard errors using k-means, $k = \min(20, \lfloor n/10 \rfloor)$, and 2000 paired resamples. REML pooling; DerSimonian–Laird pooling as sensitivity.	Hartung–Knapp inference and prediction intervals. Within-patient dependence between sections is not modeled.
**Influence analysis: LOPO/LOSO**	Sensitivity to omission of individual patients or sections.	Patient or section omitted in turn.	2,535 scheduled refits across 39 estimable readout–model combinations.	Stability follows the specified sign and magnitude criteria and their denominators. This is influence analysis, not a collinearity assessment.
**Label and depth sensitivity**	Dependence on classification thresholds, classifier genes,	Spots within the section structure; configuration or	A 770-configuration sweep; a 12-configuration battery; 5,000 within-section	Retention definitions and valid-fit denominators remain analysis-specific.

	and depth adjustment.	repeat as the sensitivity unit.	and 2,000 global permutations; gene dropout with 20 repeats per fraction.	Sensitivity runs are not independent replications.
**Unsupervised concordance**	Agreement between unsupervised partitions and operational D/E labels.	Cold spots, D + E + Ambiguous_Cold; ARI evaluated against D/E labels.	Leiden clustering on 3,000 HVGs across preprocessing variants; maximum ARI over eight resolutions compared with within-section label permutations.	Operational signal criterion: ARI ≥0.10 and $p<0.0125$, accounting for four comparators. Concordance with operational labels is not independent biological validation.
**Equivalence assessment**	Whether an interval lies within a specified practical-equivalence margin.	Unit and estimator of the corresponding analysis.	Effect-size equivalence bounds of ±0.2 and ±0.5; correlation bounds of ±0.10 where evaluated.	TOST, α=0.05: the 90% interval must lie entirely within the bounds. A 95% interval entirely beyond a margin supports the corresponding larger-than-margin classification; otherwise, the result is inconclusive. Nonsignificance is not equivalence.
**C-SIDE**	Pooled, cell-type-conditioned expression contrast, E=1 versus D=0.	Pooled spots retained by each arm; no patient or section term.	RCTD or A0 weights, each with and without section 38. Primary and arm-specific secondary cell-type sets; input and modeled spot counts differ.	Estimability requires finite σ, q, and SE, with SE<10. Holm correction uses original p-values among estimable gene–cell-type pairs within family, arm, and analysis set. No patient-level replication is inferred.
**Annotated composition: P9**	$\delta(c) = f(c,E) - f(c,D)$, where f is the annotated tumor/(tumor + stroma) spot fraction.	Cancer capture; at least 10 tumor-plus-stroma spots per label.	Primary dictionary plus sensitivity dictionaries excluding either in situ or immune stroma. Saturated captures are retained.	Median δ; 10,000 capture-bootstrap draws; exact two-sided sign test excluding δ = 0. Measures annotated spot proportions, not cell counts or tissue area.
**Annotated-interface proximity**	$AUC = P(d_A < d_{D+E}) + 0.5(\text{tie})$, where d is the hexagonal-grid distance to the nearest interface-marked spot.	Donor; one capture per evaluable donor.	Primary V0/C0 comparison: tumor versus tumoral stroma + fibrosis + immune stroma; Ambiguous_Cold versus pooled D+E. Eligibility and distance definitions are provided in note f.	Bootstrap interval for the median across units; exact sign test relative to AUC=0.5. Wilcoxon signed-rank testing was also computed. The interface dictionary differs from P9.
**Mixed-label neighborhoods**	$S_{mix} = \sum n_{mixed} / \sum n_{ambiguous}$, where mixed Ambiguous_Cold	Section-specific six-nearest-neighbor graph; global statistic	N1: within-section label permutation. N2: rigid relocation of contiguous ambiguous	$p = (1+b)/(1+B)$, where B is the number of valid null replicates and b counts null

	spots have at least one D and one E neighbor.	weighted by ambiguous-spot counts.	components onto existing spot sites. A total of 999 replicates per null.	statistics at least as extreme as observed. N1 and N2 preserve different structures; neighborhood enrichment does not validate a histological boundary.
**Compositional intermediacy**	Whether the ambiguous-group mean lies strictly between the D- and E-interior means.	Section; at least five finite observations per group and measurement.	CAF, Tumor, PDGFRB, COL3A1, COL5A1, and COL6A1. D interiors have no E neighbor; E interiors have no D neighbor.	Exact ties are non-intermediate. Section criterion: ≥4/6 measurements; cohort criterion: ≥70% of sections. This compares group means, not a continuous spatial profile.
**Pseudo-spot stress test**	Behavior of M0/M3 under composition and source-cell-type expression perturbations.	Simulation replicate; pseudo-spots in 43 pseudo-sections generated from a fixed Eval-cell pool.	Patient-level Ref/Eval split; Eval cells sampled with replacement. Target of 30 cells per spot, not a fixed total. S1: 50 replicates; S2: 30 per $\lambda \in \{1.5,2,3\}$ S3: 30 at λ = 2.	On the primary normalized scale, the unweighted mean of gene-specific proportions in S1 and gene-by-λ proportions in S2 must each reach 90%, using accepted replicates as denominators. S1 assesses intervals containing zero; S2 assesses ATT coverage; S3 comparisons are descriptive.
**Drug–gene annotation audit**	Curation and prioritization of existing interaction annotations.	Database row and normalized gene–drug pair.	Descending has_mechanism sorting before deduplication, using the implementation's default pandas sort order. Prioritization incorporates recorded interaction types, approval status recorded in DGIdb, interaction score, and an eight-gene whitelist.	Descriptive audit without inferential testing. Recorded approval does not establish a TNBC indication, and an interaction annotation is not evidence of efficacy.

Notes to Supplementary Table S2

a. Regional-model structure. The group indicator G equals 1 for the first-named group. Fixed effects include G, its section mean, within-section covariates, and their section means. Specification B includes a patient intercept and independent section-intercept and section-slope variance components; section intercept–slope correlation is not estimated. Specification A estimates correlated capture/section intercepts and slopes. Models use REML; spatial covariance between adjacent spots is not explicitly specified.

b. Multiplicity. Regional Holm families are defined separately for the reported contrast/model/constructor combinations: Family A, eight immune readouts; Family B, five E−D readouts in M0, comprising CAF abundance and four genes; the four-gene stromal family, m=4m=4m=4 across genes separately for E−D and E−I in M0/M0adj; Family C, three MYC readouts for D−I; and panel comparisons D1–D4, m=4. Non-estimable regional entries retain p=1 as a bookkeeping

placeholder, not a test result. C-SIDE correction instead includes present, estimable gene–cell-type pairs; nominal, evaluated, and estimable family sizes are distinguished.

c. Bootstrap inference. The bootstrap tail summary is

$$P_{boot} = \min\left\{1, \frac{2 \min\,(k_{\le 0}+1, k_{\ge 0}+1)}{B_{valid}+1}\right\},$$

where $k_{\le 0}$ and $k_{\ge 0}$ count accepted coefficients at or below and at or above zero, respectively, and B_{valid} is the number of accepted draws. The percentile interval uses the same accepted coefficients; zero is not imposed on the resampling distribution. Labels, expression scores, deconvolution estimates, and PCA transformations/loadings remain fixed. The >30% failure criterion is operational; a lower failure rate does not guarantee absence of selection among accepted draws.

d. Unsupervised preprocessing. Pipeline P4 combines spatial smoothing of expression with Harmony. The smoothing kernel and weights and the implementation of P3 are not specified in the available implementation records. The six-nearest-neighbor graph used for neighborhood analysis does not define the smoothing procedure.

e. C-SIDE implementation. Analyses used spacexr 2.2.1 and R 4.3.3, with doublet_mode=FALSE, cell_type_threshold=125, weight_threshold=0.8, fdr=0.01, and seed 1234. The installed default gene_threshold of 5×10^{-5} was retained. Input/modeled spot counts were: RCTD, 14,884/14,602; RCTD without section 38, 14,715/14,462; A0, 14,884/10,861; and A0 without section 38, 14,715/10,842. For 36 exported genes absent from .X, counts were reconstructed from log-normalized .raw using estimated spot totals. Accordingly, the input matrix was not composed exclusively of original counts. Here, σ denotes the natural-log fold change for E versus D, and q denotes the C-SIDE BH-adjusted p-value within cell type.

f. Annotated composition and interface. P9 defines Tumor as tumor + tumor_incierto + in_situ and Stroma as estroma_tumoral + fibrosis + estroma_inmune; other categories are excluded from the denominator. A capture is saturated when both label-specific tumor fractions equal zero or both equal one. P9 uses seed 42 and 10,000 capture-bootstrap draws.

The V0 interface dictionary defines tumor as positive and estroma_tumoral + fibrosis + estroma_inmune as negative. Eligibility requires at least 10 positive, negative, interface, Ambiguous_Cold, and comparator spots. The primary analysis includes seven evaluable captures—M2, M3, M4, M5, M6, M14, and M16—from seven donor labels. Interface inference uses seed 42 and 10,000 bootstrap draws. Neighbors of grid position (r,c) have offsets (0,±2) and (±1,±1); only spots in the classified object are considered. The hexagonal-grid distance to an interface-marked spot is

$$d = \max\left(|\Delta r|, \left\lceil\frac{|\Delta r| + |\Delta c|}{2}\right\rceil\right).$$

The exact rule generating the interface flags is not specified in the available implementation records.

g. Pseudo-spot generation and evaluation. Perturbation targets are COL3A1, COL5A1, COL6A1, and PDGFRB from CAF; MYC from Tumor; and CXCL9 from Macrophage. M3 uses true realized composition in simulation but estimated composition in real data. Per-type counts are

$$c_t = \max[round(30p_t + \varepsilon_t), 0], \quad \varepsilon_t \sim \mathcal{N}(0, 0.5^2),$$

with an independent draw for each type and p_t the normalized A0-derived proportion. If all 15 counts are zero, one randomly selected type receives 30 cells. Cells are sampled with replacement from the corresponding Eval-cell pool; Ref profiles are used for verification, not spot generation.

The primary criterion uses COL3A1, COL5A1, COL6A1, and PDGFRB. Wilson intervals describe gene-level proportions; Monte Carlo percentile intervals use 10,000 replicate resamples. ATT and the M3 coefficient need not coincide in S3. No depth-matching thinning is performed, and a degenerate Monte Carlo interval does not establish certainty of perfect coverage.

h. Drug–gene annotation. The whitelist comprises CD47, ITGA5, ITGAV, FAP, EZH2, DNMT1, PDGFRB, and TGFB1. has_mechanism indicates a recorded interaction type, not a mechanism established in TNBC. Compound-name consolidation after removal of salt terms is mechanical. Annotation counts are descriptive and are not interpreted as comparative therapeutic efficacy.

i. Model fitting. Acceptance requires convergence, finite coefficient and standard error, a retained random slope, and positive residual and slope variances. The primary fitting ladder is rescaled/centered L-BFGS → BFGS → conjugate gradient → original-scale fallback. Influence analyses and other sensitivities retain their procedure-specific fitting settings.

j. Computational environments. HPC analyses used the following environments:

- tnbc_spatial: Python 3.10.18; scvi-tools 1.1.2; cell2location 0.1.5; torch 2.6.0+cu118; pytorch-lightning 2.6.0; lightning 2.1.4; Scanpy 1.9.8; anndata 0.10.8; NumPy 1.23.5; pandas 2.0.3; SciPy 1.10.1; statsmodels 0.14.0.
- spatial_tnbc_a: Python 3.10.18; NumPy 2.2.6; pandas 2.3.3; SciPy 1.15.2; anndata 0.11.4; statsmodels 0.14.5; Scanpy 1.11.4.
- r_spacexr: R 4.3.3; spacexr 2.2.1.

Annotated-composition analyses and the drug–gene annotation audit were executed locally using Python 3.13.9, NumPy 2.3.5, and pandas 2.3.3; annotated-composition analyses additionally used SciPy 1.16.3.

k. Deconvolution settings. A0 uses posterior mean abundances stored in the discovery object. The project configuration specifies N_cells_per_location=30, detection_alpha=200, 30,000 epochs, full-batch training, learning rate 0.002, seed 42, and 1,000 posterior samples. These values describe the project configuration; their use in the A0 training execution was not independently established. A0 was not retrained for the sensitivity analysis. A3 used RNAStereoscope for 400 epochs with batch size 2,500 and SpatialStereoscope for 3,000 epochs, with seed 42 and other arguments at their installed defaults.

Abbreviations: A, Ambiguous_Cold; ARI, adjusted Rand index; ATT, average effect on E-like pseudo-spots; AUC, probability-based discrimination measure defined above; BH, Benjamini–Hochberg; CAF, cancer-associated fibroblast; DL, DerSimonian–Laird; FDR, false discovery rate; HPC, high-performance computing; HVG, highly variable gene; LOPO/LOSO, leave-one-patient/section-out; MC, Monte Carlo; PC/PCA, principal component/analysis; REML, restricted maximum likelihood; SE, standard error; TOST, two one-sided tests. No adjusted contrast or sensitivity result establishes causality or complete removal of compositional confounding.

Supplementary Table S3. Operational-label and regional-contrast sensitivity. Sensitivity of the operational labels and of the reference E − D contrast in the discovery dataset (GSE210616; 43 sections, 22 patients). Rows summarize the classifier-threshold sweep, within-section label permutation, random removal of classifier-signature genes, the twelve-configuration labeling battery, and unsupervised clustering under five pipelines. Effects are spot-level d and section-paired d_z of the reference contrast; retention is the configuration effect divided by the baseline effect.

**Analysis**	Setting	Metric	Result	Null or reference	Note
Threshold sweep	Reference configuration C0 (production percentiles 60/75, margin 0.10)	Reference E − D effect	Spot-level d 0.487; section-paired d_z 1.207	—	43 paired sections; 9,302 D and 5,582 E spots
**Threshold sweep**	770 valid of 770 planned configurations; labels re-derived	Spot-level d: sign preserved; retention ≥ 0.8; retention ≥ 0.5	100.0%; 99.1%; 100.0%	Median retention 1.02 [Q25 0.93, Q75 1.10]; P5–P95 0.84–1.19	Denominator = 770 valid configurations
**Threshold sweep**	770 valid of 770 planned configurations; labels re-derived	Section-paired d_z: sign preserved; retention ≥ 0.8; retention ≥ 0.5	100.0%; 79.1%; 100.0%	Median retention 1.00 [Q25 0.82, Q75 1.08]; P5–P95 0.71–1.17	Denominator = 770 valid configurations
**Label permutation**	Within section, among cold D/E spots (14,884 spots); 5,000 permutations	Spot-level d	Observed 0.487	Null mean ± SD 0.0113 ± 0.0129; P2.5–P97.5 −0.014 to 0.037	p = 2.00e-04 (minimum attainable 2.0e-04)

**Label permutation**	Within section, among cold D/E spots; 5,000 permutations	Section-paired d_z	Observed 1.207	Null mean ± SD −0.0021 ± 0.1578; P2.5–P97.5 −0.305 to 0.306	p = 2.00e-04 (minimum attainable 2.0e-04)
**Signature-gene dropout**	50% of classifier-signature genes removed; 20 repeats; production scorer	Exact label agreement; ARI vs stored labels	65.2%; 0.374	0% control: 100.0%; 1.000	Mean empty signatures 0.10
**Signature-gene dropout**	50% removed; 20 repeats	Retention of spot-level d; of section-paired d_z	0.84; 0.94	Baseline = 1	Repeats with d retention ≥ 0.8 / ≥ 0.5: 75% / 95%
**Twelve-configuration battery**	C0–C11: tumor ±10%/±20%, CD8 ±10%/±20%, margin ±20%, per-section quantiles; 7 central and 5 sensitivity configurations	Label counts across configurations	D 6,519–11,889; E 3,946–7,329 spots	C0: D 9,302; E 5,582	Readout-specific results are in Supplementary Tables S4, S8 and S9
**Clustering P0**	Global scaling (replica of the original method)	Best ARI vs D/E labels (resolution, k)	0.0019 (0.1, k = 21)	Null P95 0.0019; p = 0.9303	ARI vs section 0.649; ARI vs patient 0.972 at the same resolution
**Clustering P1**	Within-section scaling	Best ARI vs D/E labels (resolution, k)	0.1418 (0.2, k = 9)	Null P95 0.0082; p = 0.0050 (minimum)	ARI vs section 0.004; signal criterion met
**Clustering P2**	Harmony (batch = section)	Best ARI vs D/E labels (resolution, k)	0.0067 (0.3, k = 17)	Null P95 0.0012; p = 0.0050 (minimum)	ARI vs section 0.405; signal criterion not met (ARI < 0.10)
**Clustering P3**	Spatial smoothing + per-section scaling	Best ARI vs D/E labels (resolution, k)	0.0266 (0.3, k = 27)	Null P95 0.0137; p = 0.0050 (minimum)	ARI vs section 0.082; signal criterion not met
**Clustering P4**	Spatial smoothing + Harmony	Best ARI vs D/E labels (resolution, k)	0.1070 (0.1, k = 2)	Null P95 0.0043; p = 0.0050 (minimum)	ARI vs section 0.000; signal criterion met

Footnote: One-at-a-time profiles of the sweep varied the tumor percentile from 40 to 90, the CD8 percentile from 50 to 95 and the ambiguity margin from 0 to 0.30; configurations were valid with ≥ 50 Desert and ≥ 50 Excluded spots. The source output does not name the readout variable of the sweep; the sweep is therefore reported as a sensitivity of the reference E − D contrast and is not attributed to a specific readout such as CAF abundance. Permutation p = (1 + number of null values ≥ observed)/(N + 1). Dropout retention values are point values; the printed bracketed intervals are not reproduced because their construction is not described. Clustering used 15,706 cold spots (9,302 D, 5,582 E, 822 Ambiguous_Cold) and 3,000 highly variable genes; ARI was evaluated on the 14,884 D/E spots; the best ARI is the maximum over eight resolutions (optimistic). The signal criterion was Bonferroni $\alpha = 0.0125$ and ARI ≥ 0.10. Per-configuration results for the 770 grid points are not available in the supplied sources.

Supplementary Table S4. Regional stromal and matrix-associated contrasts. Estimated CAF abundance and four stromal genes outside the classifier signatures in the discovery dataset (GSE210616; 5,582 E, 9,302 D and 4,080 I spots; 43 sections, 22 patients), with the sensitivity analyses cited in Results and a CAF sensitivity in the secondary dataset (GSE213688, all 15 captures). The metric and the interval type are stated in each row; hierarchical estimates are d_intra with model-based 95% CIs.

Readout	Contrast	Model or population	Metric	Estimate [interval]	Adjusted p or evidence	Note
CAF abundance	E – D	M0, spec B	d_intra [95% CI]	0.777 [0.552, 1.002]	Holm p = 1.29e-11 (Family B, m = 5)	—
**CAF abundance**	E – D	43 sections	Section-paired d_z [section-bootstrap interval]	1.207 [0.980, 1.627]	42/43 sections positive; Wilcoxon p = 4.32e-12	—
**CAF abundance**	E – D	22 patients	Patient-level d_z [patient-bootstrap interval]	1.312 [1.036, 1.895]	22/22 patients positive	—
**COL3A1**	E – D	M0, spec B	d_intra [95% CI]	1.034 [0.911, 1.157]	Holm p = 1.72e-60 (Family B, m = 5)	—
**COL5A1**	E – D	M0, spec B	d_intra [95% CI]	0.682 [0.569, 0.796]	Holm p = 1.03e-31 (Family B, m = 5)	—
**COL6A1**	E – D	M0, spec B	d_intra [95% CI]	0.543 [0.459, 0.628]	Holm p = 7.88e-36 (Family B, m = 5)	—
**PDGFRB**	E – D	M0, spec B	d_intra [95% CI]	0.450 [0.376, 0.525]	Holm p = 4.02e-32 (Family B, m = 5)	—
**COL3A1**	E – D	M0adj	d_intra [95% CI]	0.908 [0.790, 1.026]	Holm p = 1.07e-50 (m = 4)	Section-bootstrap β interval [0.5539, 0.6893]
**COL5A1**	E – D	M0adj	d_intra [95% CI]	0.615 [0.517, 0.713]	Holm p = 3.83e-34 (m = 4)	Section-bootstrap β interval [0.2660, 0.3470]
**COL6A1**	E – D	M0adj	d_intra [95% CI]	0.506 [0.419, 0.593]	Holm p = 3.84e-30 (m = 4)	Section-bootstrap β interval [0.2467, 0.3308]
**PDGFRB**	E – D	M0adj	d_intra [95% CI]	0.411 [0.346, 0.477]	Holm p = 3.83e-34 (m = 4)	Section-bootstrap β interval [0.1251, 0.1698]
**COL3A1**	E – I	M0adj	d_intra [95% CI]	0.298 [0.222, 0.374]	Holm p = 5.51e-14 (m = 4)	Section-bootstrap β interval [0.1702, 0.2892]
**COL5A1**	E – I	M0adj	d_intra [95% CI]	0.217 [0.144, 0.289]	Holm p = 9.45e-09 (m = 4)	Section-bootstrap β interval [0.0885, 0.1699]
**COL6A1**	E – I	M0adj	d_intra [95% CI]	0.168 [0.119, 0.218]	Holm p = 9.21e-11 (m = 4)	Section-bootstrap β interval [0.0768, 0.1418] (24% failed replicates)
**PDGFRB**	E – I	M0adj	d_intra [95% CI]	0.121 [−0.107, 0.349]	Holm p = 0.2990 (m = 4)	Section bootstrap NI (44% failed replicates)
**CAF abundance**	E – D	Leave one patient out (22 patients)	Section-paired d_z without one patient [min, max]	1.161 to 1.335 (full data 1.207)	Maximum relative change 10.7% (patient 11); sign preserved 100.0%; half-subsample median 1.247 [P2.5–P97.5 0.945, 1.634]	Hierarchical M0 coefficient: maximum change 10.8% over 65 patient or section exclusions

**CAF abundance**	E − D	Configurations C0–C11, M0	Sign of d_intra; max/min of d_intra	Positive in 7/7 central and 5/5 sensitivity configurations; max/min 1.25	Stability rule met	—
**PDGFRB**	E − D	Configurations C0–C11, M0	Sign of d_intra; max/min of d_intra	Positive in 7/7 central and 5/5 sensitivity configurations; max/min 1.11	Stability rule met	—
**CAF abundance**	E − D	Secondary dataset, 15 captures	Capture-paired d_z [capture-bootstrap interval]	1.508 [1.036, 2.626]	14/15 captures positive; Wilcoxon p = 1.22e-04	Cancer-only CAF contrast NR
**CAF abundance**	E − D	Four deconvolution specifications, M0	d_intra by specification	A0_joint_a200 (Cell2location) 0.777; A1_solo_disc (Cell2location) 0.826; A2_joint_a20 (Cell2location) 1.174; A3_stereo (Stereoscope) 1.052	Nominal p ≤ 1.29e-11 for all four	CIs for A1–A3 NR

Footnote: d_intra = model coefficient divided by the residual SD of the same mixed model (specification B: patient > section > spot, random slope, Mundlak terms). M0adj adjusts for log UMI and estimated tumor abundance; no M0adj model was fitted for CAF abundance. Family B Holm (m = 5) covers CAF and the four genes under M0; the four-gene Holm (m = 4) is computed per direction (E − D or E − I), so PDGFRB has distinct Holm p-values in the two families. Section bootstrap: 1,000 planned replicates, resampling sections with replacement; the number of distinct patients varies between replicates (not a patient bootstrap); intervals are bootstrap 95% intervals of β (original scale), and cells with > 30% failed replicates are NI. Percent changes are relative changes of the stated statistic.

Supplementary Table S5. Selected cell-type-conditioned C-SIDE estimates. Pooled C-SIDE estimates of E- versus D-labeled expression within selected cell types in the discovery dataset, for four related arms that differ in per-spot weights (RCTD or Cell2location A0) and in the exclusion of section 38. Each cell gives σ (natural-log fold change, E versus D) with the Holm-adjusted p-value on original C-SIDE p-values. Rows include the four stromal genes within CAFs and HLA-A and B2M in the immune compartments cited in Results.

Gene	Cell type (cell-type set)	rctd	rctd_no38	a0	a0_no38
COL3A1	CAF (primary)	0.273 (2.03e-39)	0.276 (1.98e-32)	0.208 (1.25e-22)	0.209 (4.28e-23)
COL5A1	CAF (primary)	0.215 (1.59e-13)	0.204 (9.19e-13)	0.289 (4.38e-16)	0.292 (2.48e-16)
COL6A1	CAF (primary)	−0.056 (0.2690)	−0.059 (0.2299)	−0.110 (0.0493)	−0.111 (0.0488)
PDGFRB	CAF (primary)	0.123 (0.0986)	0.060 (0.2692)	0.331 (3.36e-05)	0.332 (3.20e-05)
HLA-A	CD8 T (primary)	—	—	−0.324 (2.03e-16)	−0.320 (5.49e-16)
B2M	CD8 T (primary)	—	—	−0.453 (3.97e-29)	−0.447 (2.29e-28)
HLA-A	Macrophage (secondary)	−0.423 (2.63e-33)	−0.420 (7.77e-33)	—	—
B2M	Macrophage (secondary)	−0.495 (5.27e-43)	−0.490 (4.33e-41)	—	—

Footnotes: σ > 0 = higher in E-labeled spots. Holm is applied within each gene family, arm and cell-type set, over estimable pairs only (SE finite and < 10). — = the cell type was not among the types modeled in that arm (rctd: CAF, endothelial, macrophage, cycling myeloid, normal epithelial, PVL, tumor; rctd_no38: the same without PVL; a0 and a0_no38: CAF, CD4 T, CD8 T, normal epithelial, PVL, cycling T, tumor). Macrophages belong to the secondary (exploratory) cell-type set. Modeled spots: rctd 14,602 of 14,884; rctd_no38 14,462 of 14,715; a0 10,861 of 14,884; a0_no38 10,842 of 14,715. For compactness, this table reports σ and Holm-adjusted p-values; standard errors, expression ratios and BH-adjusted q-values are not displayed. The arms share data and are not independent replicates; the model is pooled, without section or spatial terms.

Supplementary Table S6. Sensitivity of annotated tumor–stroma composition. Summaries across secondary Cancer captures (GSE213688; one capture per donor label) of δ = f(E) − f(D), where f is the fraction of tumor-annotated spots among tumor- plus stroma-annotated spots within each operational label. Rows give the primary dictionary and two alternative dictionaries, each for all evaluable captures and for non-saturated captures. Main-text Table 2 gives the per-capture values of the primary dictionary.

Dictionary	Capture set	Captures (n)	Median δ [95% capture-bootstrap interval]	δ < 0 / δ > 0 / δ = 0	Sign-test p (minimum attainable)
Primary	All evaluable	11	−0.141 [−0.335, −0.107]	9 / 0 / 2	0.0039 (0.0039)
**Primary**	Non-saturated	9	−0.248 [−0.353, −0.138]	9 / 0 / 0	0.0039 (0.0039)
**Without in situ**	All evaluable	11	−0.138 [−0.248, 0.000]	7 / 0 / 4	0.0156 (0.0156)
**Without in situ**	Non-saturated	7	−0.141 [−0.302, −0.138]	7 / 0 / 0	0.0156 (0.0156)
**Stroma without immune stroma**	All evaluable	11	−0.141 [−0.302, −0.107]	9 / 0 / 2	0.0039 (0.0039)
**Stroma without immune stroma**	Non-saturated	9	−0.227 [−0.348, −0.138]	9 / 0 / 0	0.0039 (0.0039)

Footnotes: δ < 0 = lower annotated tumor fraction in E-labeled spots. Primary dictionary: tumor = tumor, uncertain tumor and in situ; stroma = tumor-associated stroma, fibrosis and immune stroma. "Without in situ" removes in situ annotations from numerator and denominator; "stroma without immune stroma" restricts stroma to tumor-associated stroma and fibrosis. Other annotation groups are excluded from the denominator; each label requires ≥ 10 spots in the denominator. A capture is saturated when neither label has stroma-annotated spots or neither has tumor-annotated spots (δ = 0 by construction). Bootstrap: 10,000 capture resamples, seed 42. Sign test: exact, two-sided, ties excluded. Fractions refer to annotated spots, not cells or area. The concordance of the classifier tumor axis with annotations is not cited in Results and is not included.

Supplementary Table S7. Mixed-label neighborhoods and annotated-interface proximity. Discovery dataset (GSE210616): fraction of Ambiguous_Cold spots with at least one D- and one E-labeled spot among their six nearest Euclidean neighbors, compared with two null models, and the compositional intermediacy of ambiguous spots by section. Secondary dataset (GSE213688): per-donor AUC for the hexagonal-grid proximity of Ambiguous_Cold versus pooled D/E spots to the annotated tumor–stroma interface, and its summary across the seven evaluable donors.

Analysis	Unit	n	Estimate	Reference interval (type)	p or criterion
**Mixed-label fraction S1 vs N1**	822 Ambiguous_Cold spots, 43 sections	330/822	0.401	0.302–0.369 (N1 null, within-section label permutation; 999 replicates)	p = 0.0010 (minimum attainable)
**Mixed-label fraction S1 vs N2**	822 Ambiguous_Cold spots, 43 sections	330/822	0.401	0.226–0.286 (N2 null, corrected rigid relocation of ambiguous components; 999 replicates)	p = 0.0010 (minimum attainable)
**Compositional intermediacy**	Sections	43	36/43 (84%) with ambiguous means between D- and E-interior means for ≥ 4 of 6 measurements	Prespecified threshold ≥ 70% of sections	Criterion met
**Interface proximity, M2**	Donor CL-like1	30 ambiguous /	AUC 0.411	—	—

		531 comparator spots			
**Interface proximity, M3**	Donor Unstable1	34 / 808	AUC 0.476	—	—
**Interface proximity, M4**	Donor Unstable2	24 / 545	AUC 0.355	—	—
**Interface proximity, M5**	Donor Unstable3	47 / 701	AUC 0.486	—	—
**Interface proximity, M6**	Donor Unstable4	19 / 599	AUC 0.573	—	—
**Interface proximity, M14**	Donor MBC_spindle2	19 / 305	AUC 0.554	—	—
**Interface proximity, M16**	Donor MBC_NST	48 / 761	AUC 0.516	—	—
**Interface proximity, summary**	Donors (one capture each)	7 donors; 221 ambiguous spots	Median AUC 0.486 (3 above, 4 below 0.5)	0.411–0.554 (bootstrap 95% interval of the median; 10,000 resamples, seed 42)	Sign-test p = 1.0000; Wilcoxon p = 0.6875 (reference 0.5)

Footnotes: S1 is weighted by the number of ambiguous spots (Σ mixed/Σ ambiguous); section-weighted (0.404) and patient-weighted (0.400) aggregations gave p = 0.0010 under both nulls. The reported ranges summarize the corresponding null distributions and are presented as reported null intervals; they are not confidence intervals. The neighbor graph used Euclidean distances in obsm['spatial'] pixels; interface distances are hexagonal-grid steps. For variant V0, tumor annotations defined the positive class, and tumor-associated stroma, fibrosis and immune stroma defined the negative class; interface spots were counted on both sides (e.g., M2: 533 interface spots, 217 positive and 316 negative). Distance = hexagonal steps to the nearest interface spot. AUC > 0.5 = Ambiguous_Cold spots closer to the interface than pooled D/E spots. No per-capture p-values were computed. The primary Cancer-only analysis included seven of 11 captures; four Cancer captures (M8, M11, M13, M15) were not evaluable under the prespecified criteria (≥ 10 spots per class, interface, ambiguous and comparator group). The four Normal captures were outside the primary population. The summary row is at donor level (one capture per donor).

Supplementary Table S8. Regional immune readouts and key sensitivity analyses. Eight prespecified immune readouts (E − D) in the discovery dataset (GSE210616) under the score_genes constructor, models M0 and M3; formal paired panel comparisons D1–D4; and summaries of the sensitivity analyses cited in Results, including the secondary dataset (GSE213688). The metric is named in each row: d_intra (hierarchical), Δ (difference between panel contrasts, z-score units), μ (meta-analytic raw mean difference) or counts of readouts.

Analysis	Readout or comparison	Metric	M0 result	M3 result	Other result	Note
Primary readout	STING (4 genes)	d_intra [95% CI]; Holm p	0.116 [−0.023, 0.255]; 0.1022	0.059 [0.010, 0.108]; 0.0756	—	M3 nominal CI excludes 0
**Primary readout**	ISG10	d_intra [95% CI]; Holm p	0.174 [0.090, 0.258]; 1.85e-04	0.095 [0.008, 0.182]; 0.0946	—	M3 nominal CI excludes 0
**Primary readout**	MHC-I 7 genes	d_intra [95% CI]; Holm p	0.374 [0.267, 0.482]; 4.76e-11	0.186 [0.082, 0.289]; 0.0027	—	—
**Primary readout**	Six-gene composite	d_intra [95% CI]; Holm p	0.320 [0.232, 0.409]; 9.74e-12	0.210 [0.114, 0.307]; 1.39e-04	—	—
**Primary readout**	4 chemokines	d_intra [95% CI]; Holm p	0.103 [0.020, 0.187]; 0.0458	0.089 [−0.004, 0.181]; 0.1215	—	—
**Primary readout**	3 chemokines	d_intra [95% CI]; Holm p	0.101 [0.015, 0.188]; 0.0458	0.073 [−0.018, 0.164]; 0.1215	—	—

**Primary readout**	HLA-A + B2M	d_intra [95% CI]; Holm p	0.438 [0.332, 0.544]; 5.14e-15	0.255 [0.161, 0.348]; 6.79e-07	—	—
**Primary readout**	MHC-I without HLA-A/B2M	d_intra [95% CI]; Holm p	0.247 [0.140, 0.354]; 2.94e-05	0.107 [0.031, 0.184]; 0.0284	—	—
**Panel comparison**	D1: six-gene composite vs 4 chemokines	Δ [95% CI]; Holm p	0.0934 [0.0540, 0.1327]; 9.80e-06	0.0409 [0.0031, 0.0786]; 0.0676	—	Lists differ by HLA-A, B2M
**Panel comparison**	D2: six-gene composite vs 3 chemokines	Δ [95% CI]; Holm p	0.0966 [0.0557, 0.1375]; 9.80e-06	0.0509 [0.0117, 0.0902]; 0.0330	—	Lists differ by CXCL11, HLA-A, B2M
**Panel comparison**	D3: six-gene composite vs HLA-A + B2M	Δ [95% CI]; Holm p	−0.0516 [−0.0868, −0.0164]; 0.0040	−0.0224 [−0.0564, 0.0116]; 0.1964	—	Lists differ by CXCL9, CXCL10, CXCL11, CCL5
**Panel comparison**	D4: MHC-I 7 genes vs MHC-I without HLA-A/B2M	Δ [95% CI]; Holm p	0.0454 [0.0310, 0.0598]; 2.78e-09	0.0281 [0.0142, 0.0421]; 3.09e-04	—	Lists differ by HLA-A, B2M
**Constructor**	Mean log-normalized expression, 8 readouts	Readouts with Holm p < 0.05	3/8	2/8	—	M0: MHC-I 7 genes, six-gene composite, HLA-A + B2M; M3: six-gene composite, HLA-A + B2M
**M3 parametrization**	M3-PCA (3 PCs) and M3-RED (5 covariates), 8 readouts	Estimable; positive sign; Holm p < 0.05	—	PCA: 8/8; 8/8; 0/8. RED: 7/8; 7/7; 3/8	—	RED Holm-significant: MHC-I 7 genes, six-gene composite, HLA-A + B2M; STING NE under RED
**Deconvolution specification**	HLA-A + B2M; 8 readouts	M3/M0 ratio of d_intra; attenuation concordance	—	—	Ratio 0.216–0.598 across four specifications; attenuation concordant for 7/8 readouts	ISG10 discordant under Stereoscope (A3)
**Control genes and depth**	Control-gene mean of score_genes, 8 readouts	E − D d_intra (M0, seed 0); within-section Spearman ρ with log UMI	−0.269 to −0.428 (Holm p < 0.05 for 8/8)	—	Median ρ over 20 seeds 0.523–0.654 (43 sections)	—
**Constructor discrepancy**	z(score_genes) − z(mean), 8 readouts	Reduction of the label coefficient after log UMI +	—	—	81%–92%	Residual interval excludes 0 for MHC-I 7 genes

		tumor adjustment				[0.0007, 0.0214] and MHC-I without HLA-A/B2M [0.0011, 0.0233]
**Control-gene seeds**	HLA-A + B2M; STING	d_intra across 20 seeds	HLA-A + B2M 0.340–0.494 (20/20 estimable, 20/20 positive); STING 12/20 estimable	—	—	Seed 0 is the primary estimate
**Meta-analysis**	8 readouts, score_genes, 43 sections	μ; I^2; 95% prediction interval; positive sections	—	—	μ > 0 for 8/8; I^2 30.2% (STING) to 87.7% (HLA-A + B2M); PI includes 0 for 8/8; 30–39 of 43 sections positive	REML with Hartung–Knapp
**Secondary dataset**	11 Cancer captures, 8 readouts (mean constructor, spec A)	Directional concordance with discovery (M0); Holm-significant readouts	8/8 directionally concordant; 0/8 Holm-significant	No Holm-significant readout; 3/8 NE (MHC-I 7 genes, six-gene composite, 4 chemokines)	—	Capture as unit; 6,254 spots
**Secondary dataset**	HLA-A + B2M, 11 Cancer captures	d_intra [95% CI]; Holm p	0.395 [0.108, 0.682]; 0.0555	0.317 [0.010, 0.624]; 0.3457	Discovery, mean constructor, M0: 0.371 [0.255, 0.486]	—
**Secondary dataset**	15 captures, 8 readouts; HLA-A + B2M	Directional concordance (M0); HLA-A + B2M d_intra [95% CI]; Holm p	7/8 directionally concordant (STING discordant); HLA-A + B2M 0.319 [0.113, 0.524]; 0.0190	HLA-A + B2M NE	—	Sensitivity set; 7,612 spots

Footnote: Discovery: 5,582 E and 9,302 D spots, 43 sections, 22 patients; specification B (patient > section > spot, random slope, Mundlak terms); score_genes with control-gene seed 0. M0, no covariates; M3, 15 Cell2location abundances; M3-PCA, three principal components of the abundances; M3-RED, tumor, CAF, total T cells, NK + NKT and endothelial cells. Holm: m = 8 per family, model and constructor (readouts); m = 4 per constructor and model (D1–D4); m = 8 per parametrization (non-estimable readouts enter with p = 1). Δ = difference between the z-scored readouts of the two panels (z within E + D). μ is a raw mean difference; sections are treated as independent units. Nominal intervals that exclude zero are not presented as Holm-significant. Counts are tallies over the complete eight-readout sets.

Supplementary Table S9. MYC-associated measurements and adjustment sensitivity. Three MYC-associated measurements in the discovery dataset (GSE210616; 9,302 Desert and 4,080 Inflamed spots; 43 sections, 22 patients) for the Desert − Inflamed contrast: unadjusted and adjusted hierarchical estimates, correlations with estimated tumor abundance, and influence of individual patients and sections. Hierarchical estimates are d_intra with model-based 95% CIs; only M0 has a Holm-adjusted p-value.

Measurement	Analysis	Metric	Estimate [interval]	p (type)	Note
MYC mRNA	M0, spec B	d_intra [95% CI]	0.330 [0.265, 0.395]	7.69e-23 (Holm, Family C, m = 3)	—
**Hallmark36**	M0, spec B	d_intra [95% CI]	0.417 [0.303, 0.531]	1.47e-12 (Holm, Family C, m = 3)	33 of 36 genes measurable
**MYC_TF_clean**	M0, spec B	d_intra [95% CI]	0.486 [0.331, 0.640]	6.85e-10 (Holm, Family C, m = 3)	Expression-derived score
**MYC mRNA**	M1 (tumor abundance)	d_intra [95% CI]	0.285 [0.220, 0.350]	5.67e-18 (nominal)	—
**Hallmark36**	M1 (tumor abundance)	d_intra [95% CI]	0.155 [0.058, 0.252]	0.0018 (nominal)	—
**MYC_TF_clean**	M1 (tumor abundance)	d_intra [95% CI]	0.179 [0.021, 0.338]	0.0267 (nominal)	—
**MYC mRNA**	M2 (tumor + CAF abundance)	d_intra [95% CI]	0.313 [0.251, 0.375]	3.93e-23 (nominal)	—
**Hallmark36**	M2 (tumor + CAF abundance)	d_intra [95% CI]	0.197 [0.104, 0.290]	3.55e-05 (nominal)	—
**MYC_TF_clean**	M2 (tumor + CAF abundance)	d_intra [95% CI]	0.226 [0.074, 0.378]	0.0036 (nominal)	—
**MYC mRNA**	M0U (log UMI)	d_intra [95% CI]	0.270 [0.212, 0.328]	6.06e-20 (nominal)	—
**Hallmark36**	M0U (log UMI)	d_intra [95% CI]	0.108 [0.043, 0.172]	0.0011 (nominal)	—
**MYC_TF_clean**	M0U (log UMI)	d_intra [95% CI]	0.134 [0.016, 0.252]	0.0256 (nominal)	—
**MYC mRNA**	Correlation with estimated tumor abundance (D + I spots)	Pooled Spearman ρ [section-bootstrap interval]	0.181 [0.056, 0.287]	—	Meta-analytic ρ 0.155; I^2 84.2%
**Hallmark36**	Correlation with estimated tumor abundance (D + I spots)	Pooled Spearman ρ [section-bootstrap interval]	0.550 [0.465, 0.625]	—	Meta-analytic ρ 0.517; I^2 89.6%
**MYC_TF_clean**	Correlation with estimated tumor abundance (D + I spots)	Pooled Spearman ρ [section-bootstrap interval]	0.578 [0.473, 0.675]	—	Meta-analytic ρ 0.637; I^2 95.3%
**MYC mRNA**	Influence	Maximum relative change: spot-level d (LOPO); section-paired d_z (LOPO); hierarchical M0 coefficient (65 exclusions)	13.9% (patient 6); 7.4% (patient 22); 6.1% (patient 12)	—	Sign preserved in all exclusions
**Hallmark36**	Influence	Same three statistics	12.5% (patient 24); 31.9% (patient 22); 7.8% (patient 22)	—	Sign preserved in all exclusions

**MYC_TF_clean**	Influence	Same three statistics	12.7% (patient 24); 64.7% (patient 22); 10.9% (patient 22)	—	Sign preserved in all exclusions

Footnote: Positive values = higher in Desert. MYC mRNA is a single gene that belongs to the classifier Silencing signature; MYC_TF_clean is an expression-derived score, not a measurement of transcription-factor activity. M1, M2 and M0U were not part of a Holm family; their p-values are nominal and no multiplicity correction is recomputed here. Correlations: pooled spot-level ϱ with section-bootstrap intervals and random-effects meta-analytic ϱ across 43 sections are different estimands. LOPO, leave one patient out (22 patients); 65 exclusions = 22 patients and 43 sections. Retention ratios are omitted because their scale is a ratio of coefficients. The target list and gene exclusions of MYC_TF_clean are NR.

Supplementary Table S10. Summary of the pseudo-spot stress test. Limited simulated evaluation of the composition-adjusted model (M3) on pseudo-spots assembled from triple-negative breast cancer single cells (GSE176078). Scenario S1 has composition differences without an introduced expression shift; S2 adds source-cell-type shifts with E-like and D-like compositions sampled from the same within-section pool; S3 uses each group's own composition. Each row names its metric and reference; intervals express Monte Carlo precision.

**Scenario and scale**	**Gene or aggregation**	**Reference**	**Accepted fits / planned**	**Metric**	**Estimate [Monte Carlo 95% interval]**
S1, normalized	Unweighted mean of 4 primary genes (rule value)	0 (no shift)	188 / 200	Proportion of M3 95% intervals containing zero	85.0% [79.5%, 90.2%]
**S1, normalized**	Weighted by accepted fits	0 (no shift)	188 / 200	Proportion of M3 95% intervals containing zero	84.57% (159/188) [79.1%, 89.9%]
**S1, normalized**	COL3A1	0 (no shift)	49 / 50	Proportion of M3 95% intervals containing zero	61.2% [47.2%, 73.6%]
**S1, normalized**	COL5A1	0 (no shift)	47 / 50	Proportion of M3 95% intervals containing zero	100.0% [92.4%, 100.0%]
**S1, normalized**	COL6A1	0 (no shift)	47 / 50	Proportion of M3 95% intervals containing zero	78.7% [65.1%, 88.0%]
**S1, normalized**	PDGFRB	0 (no shift)	45 / 50	Proportion of M3 95% intervals containing zero	100.0% [92.1%, 100.0%]
**S1, raw**	Unweighted mean of 4 primary genes	0 (no shift)	174 / 200	Proportion of M3 95% intervals containing zero	98.9% [97.2%, 100.0%]
**S1, normalized and raw**	M0 (no composition adjustment), 4 primary genes	0 (no shift)	200 / 200 on each scale	Proportion of M0 fits rejecting zero	100.0% on each scale (degenerate interval)
**S2, normalized**	Unweighted mean of 4 primary genes and 3 λ (rule value)	Defined ATT on E-like pseudo-spots	333 / 360	Coverage relative to the defined ATT	100.0% [100.0%, 100.0%] (degenerate); M3 power over planned replicates 92.5% [89.7%, 95.0%]
**S2, raw**	Unweighted mean of 4 primary genes and 3 λ	Defined ATT on E-like pseudo-spots	331 / 360	Coverage relative to the defined ATT	100.0% [100.0%, 100.0%] (degenerate); M3 power over planned replicates 91.9% [88.9%, 94.7%]
**S3, normalized**	Unweighted mean of 4 primary genes	ATT reference (different estimand)	113 / 120	Descriptive proportion of intervals containing the ATT reference	55.7% [47.9%, 63.7%]

**S3, raw**	Unweighted mean of 4 primary genes	ATT reference (different estimand)	107 / 120	Descriptive proportion of intervals containing the ATT reference	13.1% [6.7%, 20.1%]

Footnote: Primary genes: COL3A1, COL5A1, COL6A1 and PDGFRB. Replicates: S1, 50; S2, 30 per λ (1.5, 2.0 and 3.0); S3, 30 (λ = 2.0); planned fits = replicates × genes (× λ in S2). Each replicate has 43 pseudo-sections and 14,884 pseudo-spots (5,582 E-like, 9,302 D-like). M3 uses the true realized composition of each pseudo-spot, whereas real-data analyses use estimated composition. ATT = mean over E-like pseudo-spots of expression with minus without the shift (same cells). The prespecified criterion (≥ 90% for S1 and S2 on the normalized scale, unweighted mean) was not met for S1. Monte Carlo intervals: Wilson per gene; 2.5th–97.5th percentiles of 10,000 replicate resamples for averages; they do not represent between-patient variation, and a degenerate interval does not imply certainty.

Supplementary Table S11. Prioritized drug–gene annotations by gene. Existing DGIdb drug–gene records retained in the prioritized table for the regional query genes, one row per gene with prioritized records, plus MYC and CTNNB1, whose absence informs the curation. The DGIdb funnel was 628 raw rows, 498 curated rows and 182 prioritized rows, all with a recorded interaction type; 20 of the 48 unique queried genes (50 nominal entries) had prioritized records. No new database queries were made.

Gene	Query list	Prioritized records	Records with recorded approval	Whitelist-only records	Note
**PDGFRB**	E	85	17	68	17 approved compound names = 13 active principles; includes an agonist (becaplermin)
**ITGAV**	E	12	1	4	—
**FN1**	E	7	1	0	—
**FAP**	E	6	0	0	—
**TGFB1**	E	5	1	4	—
**TGFB2**	E	5	1	0	—
**ITGA5**	E	4	0	0	—
**COL1A1**	E	2	2	0	—
**COL1A2**	E	2	2	0	—
**COL3A1**	E	2	2	0	—
**COL5A1**	E	2	2	0	—
**COL6A1**	E	2	2	0	—
**VIM**	E	1	0	0	—
**EZH2**	D	17	2	1	2 approved names = 1 active principle
**STAT3**	D	9	0	0	—
**TBK1**	D	8	1	0	—
**DNMT1**	D	6	2	2	—
**CD47**	D	3	0	0	—
**CGAS**	D	3	0	0	—
**ISG15**	D	1	1	0	Approved record (irinotecan) flagged for manual review in the source
**MYC**	D	0	0	0	73 curated rows, none with a recorded interaction type
**CTNNB1**	D	0	0	0	32 of 33 curated rows without a recorded interaction type
**Total (20 genes with records)**	E 13 genes; D 7 genes	182 (E 135; D 47)	37 (E 31; D 6)	79	168 unique compound names (E 121; D 47; none shared between lists)

Footnotes: Records are unique gene–drug rows; compound names are DGIdb drug names; active principles result from a mechanical removal of salt words, and names are not summed across genes. Recorded approval is the drug_approved field of DGIdb, the registration status of the compound without indication; it does not establish approval in triple-negative

breast cancer, efficacy or region-specific response. Interaction types are not all inhibitory. The eight-gene whitelist (CD47, ITGA5, ITGAV, FAP, EZH2, DNMT1, PDGFRB, TGFB1) retains every row with a recorded interaction type; other genes keep only approved rows or rows with interaction score > 1. Whitelist-only records entered without approval and with score ≤ 1; 68 of the 79 are PDGFRB records. E/D list assignment is query-list membership, not pharmacological selectivity. The DGIdb release and query date are NR.

Supplementary Figure S1. Analytical structure and data flow of the TNBC spatial-transcriptomic reanalysis. Datasets (discovery GSE210616, secondary GSE213688 and single-cell reference GSE176078), operational labeling, regional models, cell-type-conditioned analysis, annotated-composition and spatial analyses, simulation and drug–gene annotation audit, with the population entering each analysis (Table 1).

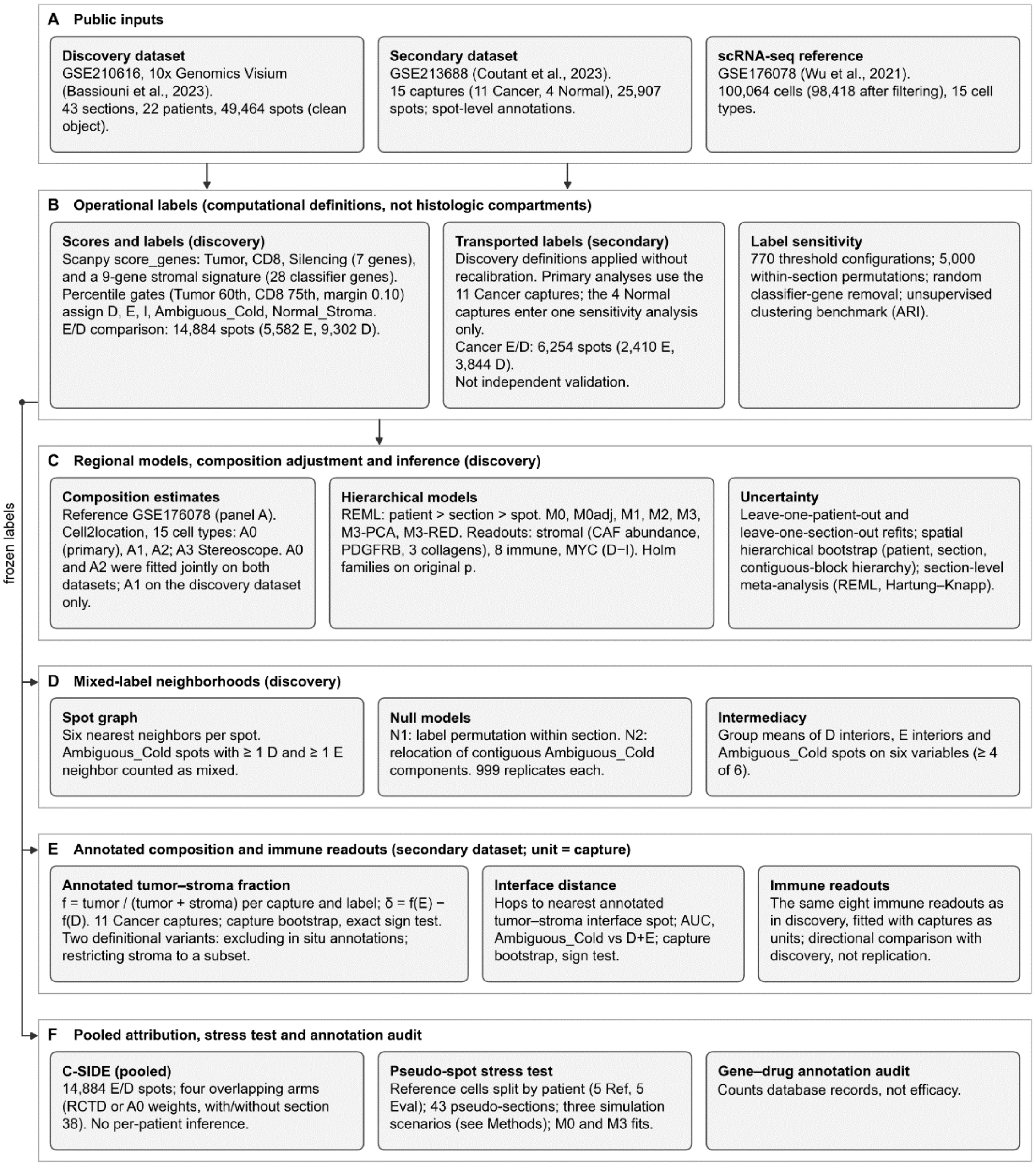

Arrows show data flow only; they do not indicate causation or biological progression. Left line: frozen labels used by D (discovery), E (transported) and the C-SIDE and pseudo-spot boxes of F; the annotation audit does not use labels. D, E, I: computationally assigned Desert, Excluded, Inflamed labels. Bulk cohorts were not analyzed. Arms and variants share data are not replications.

Supplementary Figure S2. Sensitivity of operational labels and regional contrasts. Discovery dataset. Retention of the reference E–D contrast of the threshold sweep (spot-level d and section-paired d_z, relative to baseline) across 770 classifier-threshold configurations with labels re-derived for each (tumor percentile 40–90, CD8 percentile 50–95, ambiguity margin 0–0.30). Also shown: within-section label permutation (5,000 permutations) and random removal of classifier genes (5–50%; 20 repeats per fraction). Exact label agreement, preservation of direction and retention of d and d_z are reported as separate quantities. Numerical values are given in Supplementary Table S3.

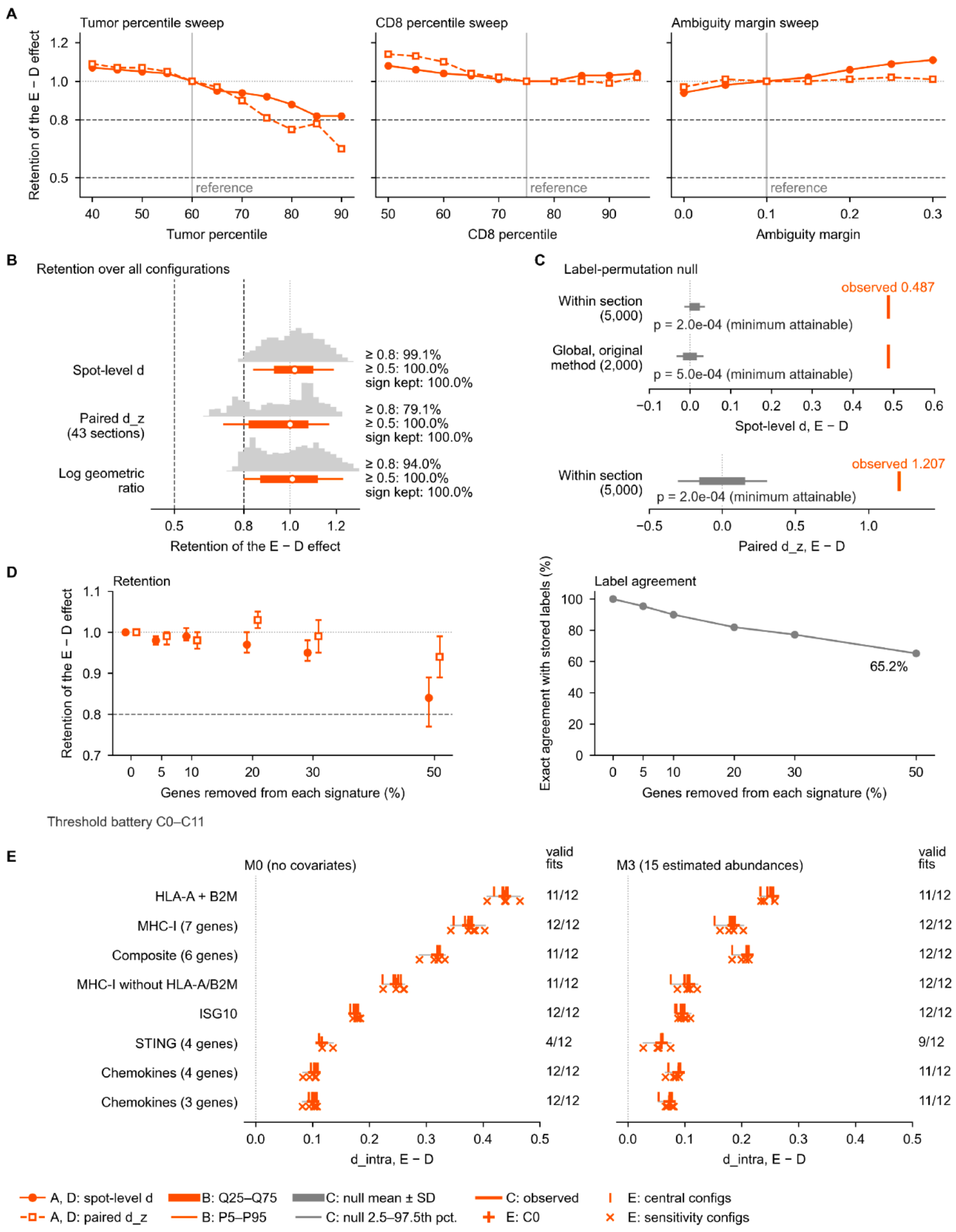

Supplementary Figure S3. Unsupervised clustering of cold spots. Leiden clustering of 15,706 cold spots (9,302 D, 5,582 E, 822 Ambiguous_Cold; 43 sections; 3,000 highly variable genes) under five preprocessing pipelines and eight resolutions. Agreement with operational labels (adjusted Rand index) was evaluated on the 14,884 D/E spots, excluding Ambiguous_Cold spots from that criterion, and was compared with the 95th percentile of within-section label-permutation nulls. Agreement with section and patient identifiers is shown for reference. Summary values are given in Supplementary Table S3.

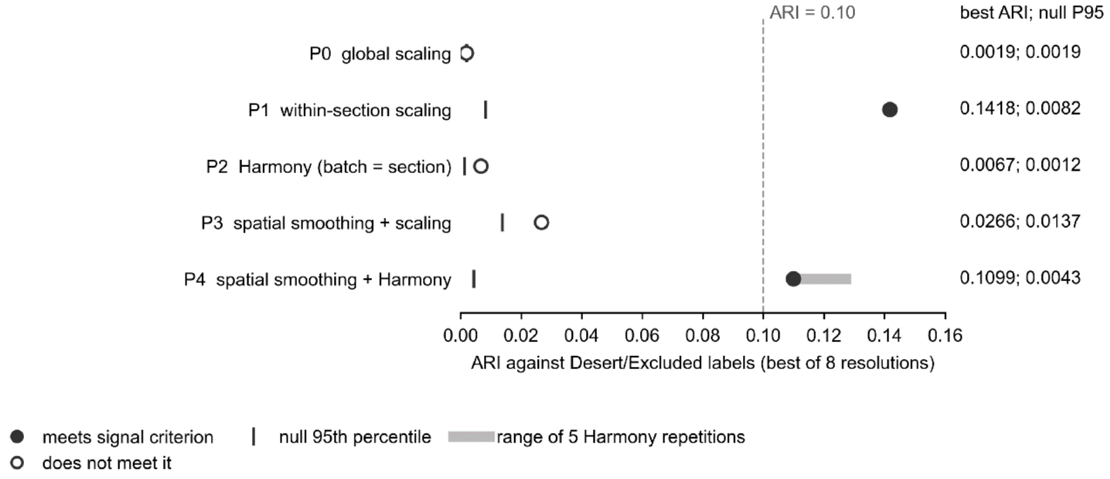

Supplementary Figure S4. MYC measures, D-labeled versus I-labeled regions. Discovery dataset (9,302 D- and 4,080 I-labeled spots; 43 sections). Standardized within-section D−I differences for MYC mRNA, Hallmark36 (33 measurable genes) and the expression-derived MYC_TF_clean score under M0 and the adjustment models shown (estimated tumor abundance, tumor plus CAF abundance, or log UMI). Leave-one-patient-out influence is shown separately for spot-level d, section-paired d_z and the hierarchical M0 coefficient. Numerical values are given in Supplementary Table S9.

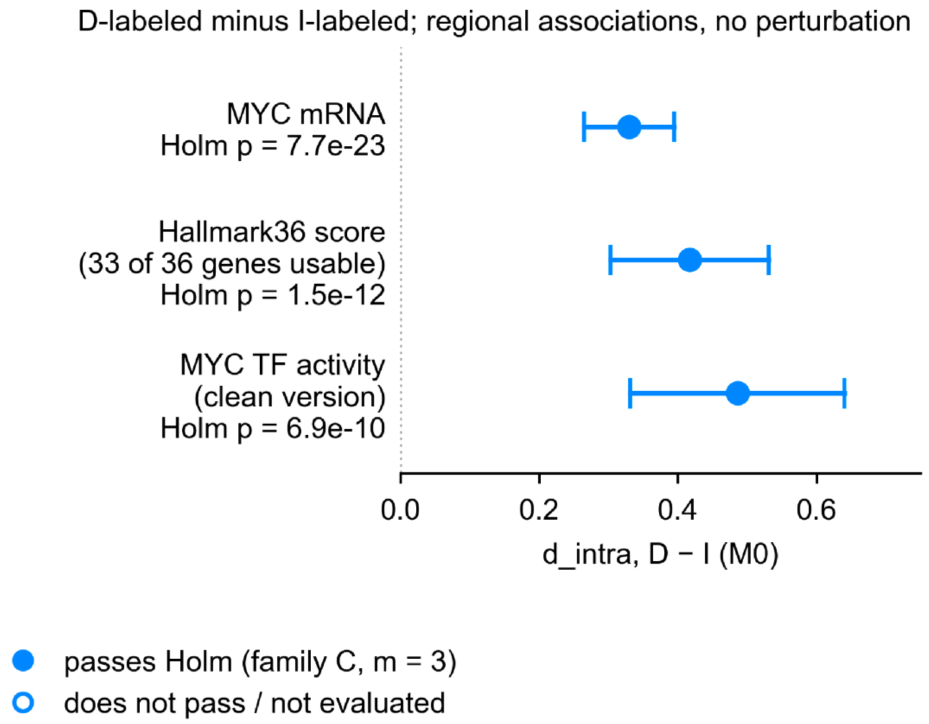

Supplementary Figure S5. Pseudo-spot stress test of the composition-adjusted model (M3). Pseudo-spots were generated from TNBC cells of GSE176078, partitioned by patient into reference (5 patients; 22,966 cells, used for verification) and evaluation (5 patients; 19,546 cells, used for sampling) sets. Each replicate comprised 14,884 pseudo-spots in 43 pseudo-sections. Per-type counts targeted 30 cells per spot; the realized number was the sum of type counts (mean 30.5, range 21–41). M3 used the true realized composition in the simulation, whereas analyses of real data used estimated composition. Scenarios: S1, composition differences without an introduced shift (50 replicates); S2, source-cell-type expression shifts ($\lambda = 1.5$, 2 and 3; 30 replicates each), with E-like and D-like compositions sampled from the same within-section pool; S3, both differences combined (λ = 2; 30 replicates). Metrics: in S1, the proportion of M3 95% intervals containing zero; in S2, the proportion containing the defined average effect on E-like pseudo-spots (ATT); in S3, a descriptive comparison of the M3 coefficient with the ATT. Gene-level intervals are Wilson intervals over accepted fits, and averages carry Monte Carlo intervals; power, where shown, is computed over planned replicates. The primary scale is the normalized scale. Numerical values are given in Supplementary Table S10.

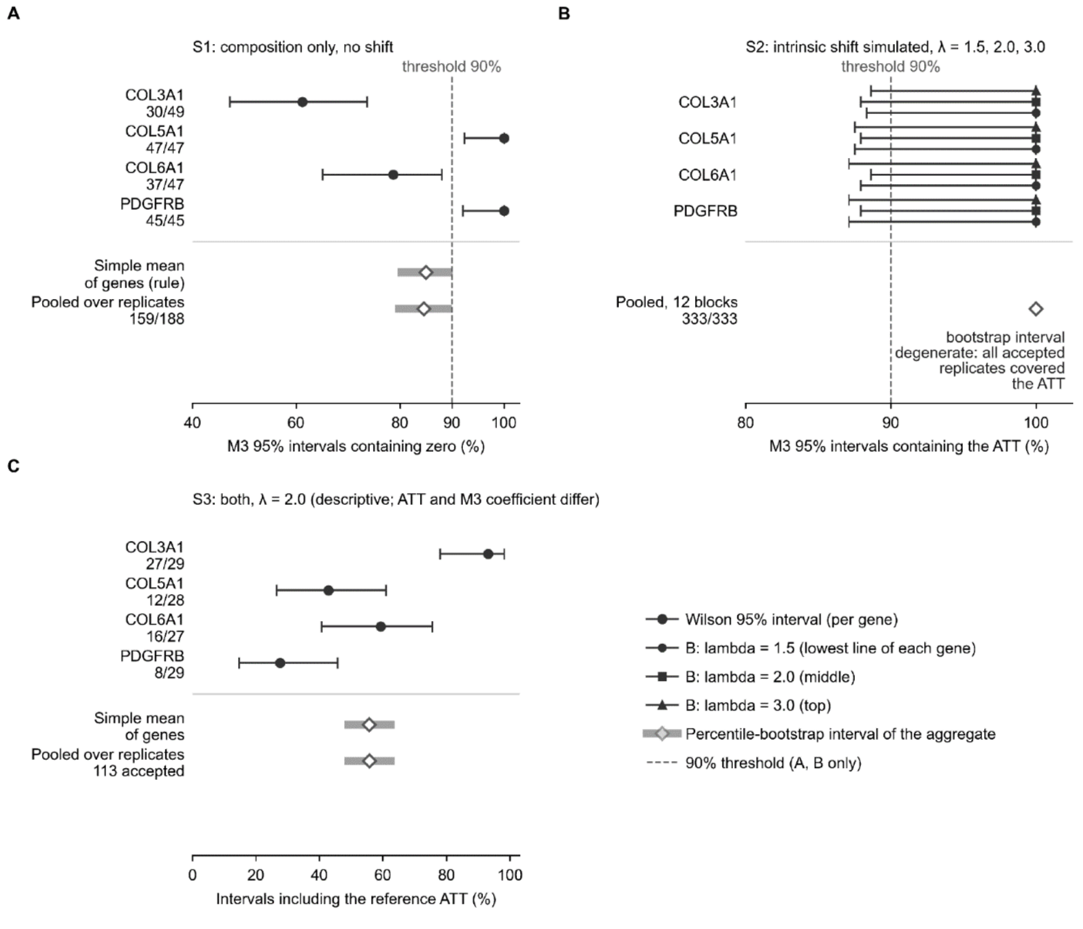

Supplement: Supplementary file 1 [file cancers-18-03476-s001.zip › cancers-4592305-supplementary.pdf]
